# The small intestine: dining table of host–microbiota meetings

**DOI:** 10.1093/femsre/fuad022

**Published:** 2023-05-16

**Authors:** Karen Delbaere, Inez Roegiers, Auriane Bron, Claude Durif, Tom Van de Wiele, Stéphanie Blanquet-Diot, Ludovica Marinelli

**Affiliations:** Center for Microbial Ecology and Technology (CMET), Department of Biotechnology, Ghent University, Coupure Links 653, Building A, 9000 Ghent, Belgium; Center for Microbial Ecology and Technology (CMET), Department of Biotechnology, Ghent University, Coupure Links 653, Building A, 9000 Ghent, Belgium; Université Clermont Auvergne, UMR 454 MEDIS UCA-INRAE, Place Henri Dunant 28, F-63000 Clermont-Ferrand, France; Université Clermont Auvergne, UMR 454 MEDIS UCA-INRAE, Place Henri Dunant 28, F-63000 Clermont-Ferrand, France; Center for Microbial Ecology and Technology (CMET), Department of Biotechnology, Ghent University, Coupure Links 653, Building A, 9000 Ghent, Belgium; Université Clermont Auvergne, UMR 454 MEDIS UCA-INRAE, Place Henri Dunant 28, F-63000 Clermont-Ferrand, France; Center for Microbial Ecology and Technology (CMET), Department of Biotechnology, Ghent University, Coupure Links 653, Building A, 9000 Ghent, Belgium

**Keywords:** small intestine, microbiota, nutrition, host-bacteria interaction

## Abstract

Growing evidence suggests the importance of the small intestinal bacteria in the diet–host–microbiota dialogue in various facets of health and disease. Yet, this body site is still poorly explored and its ecology and mechanisms of interaction with the host are just starting to be unraveled. In this review, we describe the current knowledge on the small intestinal ecology, its composition and diversity, and how the intestinal bacteria in homeostatic conditions participate in nutrient digestion and absorption. We illustrate the importance of a controlled bacterial density and of the preservation of absorptive surface for the host’s nutritional status. In particular, we discuss these aspects of the small intestinal environment in the framework of two disease conditions, namely small intestinal bacterial overgrowth (SIBO) and short bowel syndrome (SBS). We also detail *in vivo, ex vivo*, and *in vitro* models developed to simulate the small intestinal environment, some applied for (diet–)host–bacteria interaction studies. Lastly, we highlight recent technological, medical, and scientific advances applicable to investigate this complex and yet understudied body environment to broaden our knowledge in support of further progress in the medical practice, and to proceed towards the integration of the (small)intestinal bacteria in personalized therapeutic approaches.

## Teaser

Updated overview on the interaction between diet, host and microbiota in the small intestine of adult humans, in healthy and disease conditions.

## Introduction

For millions of years, resident microbes have been coevolving with their host, establishing highly specialized ecological niches and a fine-regulated cross-talk in distinct body sites, and continuously shaping homeostasis for both the host and the gut ecosystem. In this way, the gut microbiota is closely associated to human health, and became the aim of intense scientific studies.

Among the body sites colonized by microbes, the digestive tract, and particularly the small intestine, is a crucial interface where the dialogue between host, microbes, and environmental factors is complex and profound. As the small intestine is the main site of nutrient digestion and absorption, it is crucial to understand how the complex cross-talk between gut physiology, dietary factors, and the small intestinal microbiota may affect host health status.

Anatomical or pathological alterations in disease conditions may alter this fine dialogue between nutrition, host, and microbe and disrupt homeostasis.

To untangle this complex interaction in health and disease, most studies relied on fecal samples to characterize the intestinal ecology, advantageous for the non-invasive collection, although unable to capture the diverse microbial phylogeny and functionalities along the gastrointestinal tract. The small intestine is in fact a poorly accessible body site, which makes the direct sampling challenging and invasive. Consequently, the microbial ecology of the different small intestinal regions remained undescribed for a long time. The expansion of omics techniques, high-throughput sequencing, and metagenomic and metabolomic approaches, have greatly expanded our knowledge in functionality and microbial composition of the small intestine. Yet, the accurate quantification and characterization of its ecology still remains limited by technique-dependent sampling bias. Additionally, studying cellular signaling mechanisms that govern the host–microbiota–diet dialogue *in vivo* is challenging and hinders the acquisition of novel insights. In this view, diverse *ex vivo* and *in vitro* approaches have been developed to obtain a more mechanistic understanding of host–microbe dialogue in the small intestine that can further complement or even support *in vivo* observations.

This literature review, therefore, aims at discussing recent insights in the description of the small intestinal ecology, epithelium, and its interaction with dietary constituents. We put particular emphasis on the impact from the host–microbiota–diet interplay in the duodenum, jejunum, and ileum on nutrient digestion and absorption under normal healthy conditions and how this is affected when ecological and/or epithelial homeostasis is disrupted in small bowel syndrome and small intestinal bacterial overgrowth (SIBO). We will cover recent breakthroughs, innovative *in vivo, in vitro*, and *ex* models and discuss potential novel scientific routes to address mechanisms of interaction that are currently poorly understood.

## The small intestine: anatomy and function in digestion and absorption

**Figure 1.**
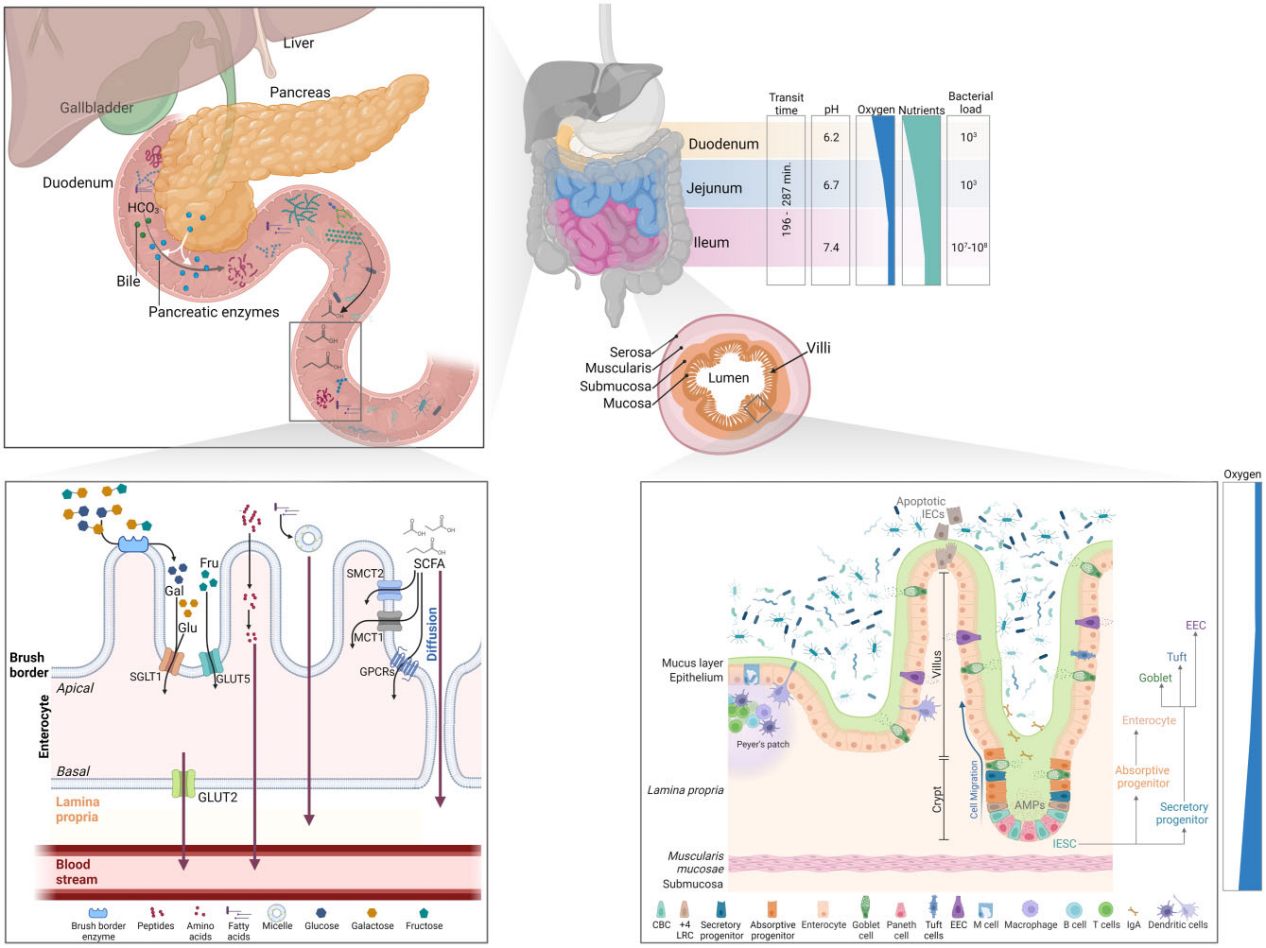
Overview of small intestinal anatomy, histology, and key processes for food digestion and absorption. Physico-chemical parameters, and bacterial load (CFU/ml) are indicated for each segment of the small intestine. pH values are based on Ibekwe et al. (2008) in fasted patients. Cell types present on the small intestinal epithelium are represented. Key digestive processes taking place in the small intestine are summarized in the top left-hand panel and, below, the major transport pathway for nutrient absorption. IESC: intestinal epithelial stem cell; IECs: intestinal epithelial cells; CBC: crypt base columnar cell; +4 LRC: +4 label retaining cell; EEC: enteroendocrine cells; IgA: immunoglobulin A; AMPs: antimicrobial peptides; Gal: galactose; Glu: glucose; Fru: fructose; SCFA: short-chain fatty acids; and GPCRs: G-protein coupled receptors. Created with BioRender.com.

Food digestion and nutrient absorption are fine-regulated processes allowing to extract energy from the diet and contribute to the maintenance of the vital functions in the human body. The main organ devoted to these functions is the small intestine, a curved tubular structure with average length of 690.1 ± 93.7 cm, forming the longest organ in the body (Tacchino 2015). The small intestine begins at the pylorus and ends at the ileocecal valve and comprises three parts: duodenum, jejunum, and ileum (Fig. 1). In the small intestine, the median transit time, guaranteed by motility patterns, such as peristalsis, segmentation and mixing, varies between 196 and 287 minutes depending on the studied group (age, gender, environmental factors, i.e. smoke) and measurement technique (device and marker) (Camilleri et al. 1991, Degen and Phillips 1996, Graff et al. 2001, Worsøe et al. 2011, Wang et al. 2015, Nandhra et al. 2020, Tominaga et al. 2020). During this time, the food (or partly digested chyme) is exposed to diverse secreted pancreatic and intestinal enzymes and physicochemical parameters existing along the gastrointestinal tract. Specific cellular receptors are also differentially expressed on the surface of the intestinal epithelium, allowing the uptake of the nutrients, available in the lumen. Overall, the different secretions and receptors define functional-specialized intestinal segments.

In these complex processes, the host is supported by the metabolic activity of the intestinal microbiota, able to break down nutrients, otherwise inaccessible to the host’s digestive enzymes. In this section, the key host’s digestive and absorption processes are explained.

### Physico-chemical parameters in the small intestine

In the small intestine, the acid chyme resulting from the passage through the stomach is discharged, then neutralized by intestinal secretion in the duodenum (Agrawal and Aoun 2014). As a result, the pH ranges from very acidic in the stomach to slightly basic (pH 7.3–7.7) in the ileum and varies from pH 5.7 to 6.4 in the duodenum and jejunum (Table 1).

**Table 1.** pH values measured with different techniques in the duodenum, jejunum, and ileum of either fasted or fed healthy adults.

		pH values			
Measurement technique	Status	Duodenum	Jejunum	Ileum	References
Double-lumen catheter	Fasted	7	6.8	No data	Moreno et al. (2006)
Double-lumen catheter	Fasted	5.88	No data	No data	Clarysse et al. (2008)
	Fed	6.1			
Radiotelemetry (1-day follow-up)	Fasted	6.5	6.8	7.2	Ibekwe et al. (2008)
	Fed	6.2	6.7	7.4	
Double-lumen catheter	Fasted	6.22	No data	No data	Riethorst et al. (2015)
	Fed	6.78			
SmartPill^TM^ (1-day follow-up)	Fasted	5.9	No data	7.5	Schneider et al. (2016)
	Fed	5.6		7.2	
Intellicap (1-day follow-up)	Both	6.8–7.2			Maurer et al. (2015)
Intellicap (1-day follow-up)	Both	Proximal: 6 (5.9–6.3); distal: 7.7 (7.4–7.8)			Koziolek et al. (2015)

Along with the pH, the presence of intestinal bacteria as well as the metabolism by host cells determine a concentration gradient of different gases (e.g. oxygen, carbon dioxide, nitrogen, and hydrogen) across the gastrointestinal tract. In particular, the concentration of carbon dioxide increases in the duodenum, before being reabsorbed in the colon (Cormier 1990). Conversely, oxygen concentration decreases from high levels in the oral compartment, to microaerophilic in the small intestine and complete anoxic conditions in colon. In humans, the oxygen tension in the small intestine and colon, measured at the serosal surface by intraoperative tissue oximetry, ranges from 36.0 ± 9.7 mmHg in the mid-ileum, to 33.5 ± 11.5 mmHg in the terminal ileum, to a minimum of 29.3 ± 11.0 mmHg in the descending colon (Sheridan et al. 1990). Moreover, an oxygen gradient exists when moving from the microaerophilic small intestinal lumen towards the highly vascularized oxygen-rich subepithelial mucosa (reviewed in Espey 2013) (Fig. 1), determining different niches for the intestinal microbes.

### Nutrient digestion in the small intestine

Through the pancreatic and bile ducts, digestive enzymes produced in the pancreas and bile from the liver, are released in the lumen of the duodenum. Here, peptides, starch, and triglycerides are broken down in smaller subunits by the action of pancreatic proteases (Ross et al. 2013), pancreatic amylases (Goodman 2010), and pancreatic lipases. Additionally, pancreatic nucleases (DNase and RNase) allow some of the nucleotide bases to be recycled and used as building blocks for human DNA and RNA synthesis (Hoard and Goad 1968). Lipids are emulsified by bile to facilitate their solubilization and further absorption in the small intestine. A minor fraction of bile salts is subjected to chemical modifications by intestinal microbiota, pivotal for the generation of secondary bile acids. As such, microbial bile salt metabolism in the small intestine and more distally in the colon may indirectly impact lipid metabolism. For example, following deconjugation by microbial bile salt hydrolase (BSH), bile salts will be less able to emulsify lipids lowering the accessibility to lipase that are essential for lipid digestion. The lipid metabolism is, thus closely linked to the small intestinal microbiota activity, for generating secondary bile acids.

Additionally, the small intestinal microbiota is involved in the cholecystokinin (CCK) hormone regulation (Martinez-Guryn et al. 2018), crucial for lipid digestion, by inducing the release of bile salts from the gallbladder and the secretion of pancreatic lipase in the small intestine. Coherently, in germ-free animals, impaired lipid digestion and downregulation of CCK are observed, compared to conventional mice (Martinez-Guryn et al. 2018). Upon administration of two bacterial strains (either live bacteria and conditioned media) in germ-free mice, an increased expression of CCK receptor (*Cckar*) in the pancreas was observed, further confirming the bacterial impact on the regulation of lipid metabolism (Martinez-Guryn et al. 2018). Lastly, LPS has been identified to bind Toll-like receptors (TLRs) expressed in enteroendocrine cells (EECs), which in turn triggers the release of CCK *in vitro* (Bogunovic et al. 2007).

The final stage of luminal digestion takes place closer to the epithelium, by the action of digestive enzymes present on small intestinal brush border of the epithelial cells. These enzymes include several oligopeptidases (i.e. aminopeptidases, carboxypeptidases, endopeptidases, and dipeptidases), lipases (i.e. sphingolipid hydrolyzing enzymes and phospholipases), and oligosaccharidases (i.e. α1,4-glucosidases, α1,6-glucosidases, α1,β2-glycosidase, β1,4-glycosidases, and α1,α1-glucosidase), required to reduce nutrients dimers and oligomers into monomers (extensively reviewed in Hooton et al. 2015).

Nondigestible carbohydrates, including mostly fibers from plants (e.g. cellulose) or other dietary sources (e.g. glycan and glycogen), are resistant to the action of the several host’s oligosaccharidases (ß-galactosidase, α-glucosidase, sucrase- α-dextrinase, and trehalase) composing the digestive enzymatic pool, yet accessible to the gut microbiota.

The bacterial metabolic activity on complex carbohydrates produces, as end by-products, short-chain fatty acids (SCFAs) of which acetate, butyrate, and propionate are the most abundant in the human small intestine. In sudden-death victims, the total SCFA concentration increased from 0.6 to 13 mmol/kg of intestinal content in the jejunum and ileum, respectively (Cummings et al. 1987), while, in ileostomy effluent, the total SCFA ranged between 51.9 and 119 mM, in slightly different ratios compared to sudden-death victims (Zoetendal et al. 2012) (Table 2). Besides SCFA, lactate (2.5–11.6 mM in ileostomy effluents), formate (0–26 mM in ileostomy effluents) (Zoetendal et al. 2012), and succinate (3.7 mmol/kg and 8.3 mmol/kg in jejunum and ileum, respectively) (Cummings et al. 1987) were detected. Lastly, the chyme is transported to the large intestine, whose primary function is to absorb water and electrolytes, and where the abundant resident microbiota continues the degradation of the nondigestible dietary fibers initiated in the terminal small intestine.

**Table 2.** SCFA concentrations in the human small intestine, measured in sudden-death victims and ileostomy effluents. Values reported in %, mM, or mmol/kg of total SCFA in intestinal content, depending on the studies.

	Sudden-death victims			Ileostomy
	*Duodenum*	*Jejunum*	*Ileum*	*Ileum*
**Acetate**	No data	0.6 mmol/kg	7.9 mmol/kg (60%)	78%
**Propionate**	No data	No data	1.5 mmol/kg (12%)	5%
**Butyrate**	No data	No data	2.3 mmol/kg (18%)	17%
**Total SCFA**	0.6–13 mmol/kg			51.9 and 119 mM
**Reference**	Cummings et al. (1987)			Zoetendal et al. (2012)

### Nutrient absorption in small intestine

Once the dietary components are broken down into monomers, an estimated 85% of them penetrate the small intestinal epithelium by diffusion, whereas the remaining 15% is taken-up by transcytosis (Szefel et al. 2015). However, numerous receptors and transport systems are localized on the epithelium, specific for the different class of molecule to absorb. Then, through underlying blood and lymphatic capillaries in the submucosa, the absorbed nutrients transit into the bloodstream.

Upon digestion in the lumen and cell intake, peptides may undergo intracellular digestion by cytosolic enzymes such as aminotripeptidases, dipeptidases, or by lysosomal and microsomal enzymes. Within minutes, virtually all the last dipeptides and tripeptides are digested in the cytosol to single amino acids, which then pass on through to the basal side of the enterocyte and then into the blood. The transfer of amino acids into and out of cells or cellular organelles is ensured by transmembrane proteins (amino acid transporters – AAT) (reviewed in Kandasamy et al. 2018).

The fate of products of fat digestion (fatty acids, monoglycerides, glycerol, cholesterol, and fat-soluble vitamins) proceeds *via* the cellular intake on the intestinal epithelium in form of micelles. Once in the cell, short- and medium-chain fatty acids and glycerol can be absorbed into the bloodstream. Conversely, long-chain fatty acids and other digestion products need to reassemble into chylomicrons before passing into the lymph vessels and, from there, being delivered in the bloodstream. In the terminal ileum, 95% of the bile acids are reabsorbed (De Aguiar Vallim et al. 2013), through a combination of passive absorption and active transport in the proximal and distal small intestine, respectively. Unconjugated bile acids are actively transported in the terminal ileum through the apical sodium dependent transporter (ASBT) and reaches the portal vein by a basolateral heterodimeric organic solute transporter (OST) (reviewed in Dawson et al. 2009). The small intestinal microbiota has also an important role in host fatty acid absorption mainly *via* some *Clostridiaceae* strains such as *Clostridium bifermentans*, able to affect the gene expression of triglycerides re-esterification enzymes, diacylglycerol O-acyltransferases (Dgat1 and Dgat2) in mice small intestine (Martinez-Guryn et al. 2018).

From the host’s enzymatic break down of digestible carbohydrates, the resulting glucose and galactose are internalized into the enterocytes by active carrier transport, through the apical sodium–glucose cotransporter 1 (SGLT1), whereas fructose pass by facilitated diffusion through the apical glucose transporter GLUT5. The absorption into the blood circulation is ensured by the transporter GLUT2, expressed on the basal side of the enterocytes (reviewed in Koepsell 2020) Conversely, from the microbial activity on nondigestible carbohydrates, SCFAs are formed. Dependent on the luminal concentration, SCFAs enter the epithelium by diffusion or through active transport systems in the apical membrane of intestinal epithelial cells (Iwanaga et al. 2006). Monocarboxylate transporter 1 (MCT1), whose expression is higher in the colon than in the small intestine (Gill et al. 2005) and sodium-coupled monocarboxylate transporter 2 (SMCT2 or SLC5A12), exclusively expressed in the small intestine, support the uptake of SCFAs and monocarboxylates such as lactate, respectively (Sivaprakasam et al. 2018). Additionally, G-protein coupled receptors (GPCRs) were also identified as receptor for SCFAs and thus, named also free fatty acid receptors (FFARs), differently binding to diverse SCFAs. In particular, GPR41 (FFAR3) and GPR43 (FFAR2), recognize acetate, butyrate, and propionate, while GPR109a (HCAR2) is solely activated by butyrate (Brown et al. 2003, Thangaraju et al. 2009). After absorption, the metabolic fate of the SCFAs differs. In mammals, butyrate is the primary energy source for colonocytes, oxidized *via* β-oxidation and tricarboxylic acid cycle. Propionate and acetate are transported to the liver and peripheral tissues, respectively and both are used as substrates for energy metabolism and lipid synthesis (Wong et al. 2006). Around 6%–9% of the total energy intake for humans, accounts from SCFA absorption (Wong et al. 2006). However, the biological effects of SCFAs are not restricted to their sole role as energy substrates for the epithelial cells, but they also contribute to water and electrolyte absorption in the colon, modulate the mucosal immune system and aid in the maintenance of the mucosal barrier (reviewed in Martin-Gallausiaux et al. 2021).

### Histology

As the major absorptive site, the small intestine has several architectural modifications of the mucosa and submucosa to increase its surface, namely folds or *plicae circularis*, arranged circularly around the lumen, and villi and microvilli, covering the apical surface of the small intestine (Fig. 1). The *plicae*, villi, and microvilli are long and numerous in the duodenum and jejunum and decrease in abundance and thickness towards the proximal ileum. Microvilli are present on the surface of each epithelial cell, packed together to form the so-called brush border, devoted to the secretion of digestive enzymes (brush border enzymes), absorption, and cellular adhesion (reviewed in Walton et al. 2016).

In the small intestinal epithelium, enterocytes are dedicated to perform terminal digestion of polysaccharides and peptides and absorb nutrients present in the intestinal lumen. This cell type, comprising for about 80% of the small intestinal epithelial cells (Van Der Flier and Clevers 2009) is characterized by a specialized portion of the cell membrane on the luminal surface, the microvillar membrane, or brush border, bearing digestive enzymes and specific carrier proteins. Along with its role in digestion and absorption, enterocytes participate in the formation of a biochemical barriers, to prevent the diffusion of pathogens, toxins, and allergens from the lumen to the mucosa (Peterson and Artis 2014). This selectively permeable barrier depends on the interaction of several barrier components, including mucus, immunoglobulin A (IgA), and antimicrobial peptide secretion, to segregate microorganisms and allergens in the lumen. In particular, enterocytes secrete several microbicidal or antiviral agents and transfer immunoglobulins from the mucosal plasma cells to the lumen.

An important component of this chemical barrier is also the mucus layer, a single thin layer covering the small intestinal epithelium, constituted by mucins, expelled through a distinctive mode of secretion referred as ‘expanding secretion’ (Dolan et al. 2022). The secreted mucus provides protection, lubrication, and hydration of the human epithelial tissues exposed to the environment (Andrianifahanana et al. 2006). In the human genome, 21 mucin (MUC) genes are known, encoding for secreted or membrane-bound mucins (Boltin et al. 2013) (summarized in Table 3). Among the secreted mucins, MUC2 is the most predominant in the small intestinal epithelium. Besides protection for the epithelium, mucins offer nutrient support for adhering bacteria, promoting their colonization of the outer part of the mucus layer (Liévin-Le Moal and Servin 2006).

**Table 3.** Characteristics and distribution of mucins in the small intestine of adult humans.

Mucins	Gel forming	Location	Cell identity	References
***Membrane-associated mucins***				
MUC1	No	Duodenum, ileum	Crypt, Brunner’s gland	Cao et al. (1997), Buisine et al. (2001), Paulsen et al. (2006)
MUC3	No	Duodenum, jejunum, ileum	Goblet cells and absorptive cells, predominance on villi	Cao et al. (1997), Williams et al. (1999a, b), Buisine et al. (2001), Paulsen et al. (2006), Audie et al. (1993)
MUC4	No	Duodenum, jejunum, ileum	Crypt, Brunner’s gland, columnar cells	Buisine et al. (2001), Paulsen et al. (2006), Audie et al. (1993)
MUC12	No	Small intestine	Enterocytes	Williams et al. (1999a), Yamamoto-Furusho et al. (2015)
MUC13	No	Duodenum, lower in ileum and jejunum	Goblet cells and columnar cells	Williams et al. (2001)
MUC17	No	Duodenum, lower in jejunum and ileum	Mature epithelial cells of the villi, enterocytes	Gum et al. (2002
***Secreted mucins***				
MUC2	Yes	Duodenum, jejunum, ileum	Goblet cells on villi and crypt	Buisine et al. (2001), Audie et al. (1993)
MUC5AC	Yes	Duodenum	Goblet cells, Brunner’s gland	Paulsen et al. (2006)
MUC5B	Yes	Duodenum	Goblet cells, Brunner’s gland	Paulsen et al. (2006)
MUC6	Yes	Duodenum	Brunner’s gland	Paulsen et al. (2006)
MUC11	Yes	Small intestine	/	Williams et al. (1999a)
MUC7	Yes	Duodenum	Columnar cells, goblet cells, Brunner’s gland	Paulsen et al. (2006)
MUC8	Yes	Duodenum	Columnar cells	Paulsen et al. (2006)

Exposed to constant environmental stimuli, the intestinal epithelium has evolved sensing strategies to detect the passage of food or the presence of potential harmful compounds and microorganisms. EECs detect luminal content and coordinate the response of the gastrointestinal tract to food ingestion, through the secretion of hormones. Present in the small intestine at a density of approximately 1%, EECs represent a family of cell subtypes classified according to their localization, shape, and hormonal secretion profile (reviewed in Guo et al. 2022). Hormones are sorted into secretory granules by carboxypeptidase E (Hosaka et al. 2005, McGirr et al. 2013) along with granins, including secretogranin III and chromogranin A, the former being extensively used as a specific marker of EEC in intestinal epithelium. EEC subpopulations express a wide range of receptors, enabling the detection of luminal content or the response to paracrine stimulation (Raybould 2010, Reimann et al. 2012), among which G-protein-coupled receptors (GPCRs). For example, through a mechanism mediated by GPR43 and inhibition of histone deacetylases, butyrate, and propionate have been described to stimulate peptide YY (PYY) expression in human EEC cell lines (Larraufie et al. 2018). Furthermore, both *in vitro* and *in vivo* studies demonstrated that EECs also express functional TLRs and respond to bacterial TLR ligands (Larraufie et al. 2017), supporting the role of EECs as sensor of gut microbiota.

Along with these cell types, sentinels driving type 2 immune mechanisms, in response to pathogens (Gerbe et al. 2012), are sporadically distributed on the epithelium and termed Tuft cells. The prevalence of this cell type in the human small intestinal epithelium has not been reported yet, but, in the human sigmoid colon Tuft cells are present at a density of ∼100 cells per square millimeter tissue (Kjærgaard et al. 2021). Yet, the presence of Tuft cells in the mouse small intestine has been documented (Banerjee et al. 2021). Additionally, it has been reported that succinate, derived from intestinal bacteria, drives the expansion of a subpopulation of Tuft cells (ATOH1-independent tuft cells) exclusively present in the small intestine, and ultimately participating in the reduction of chronic intestinal inflammation in mice (Banerjee et al. 2021).

Invaginations of the mucosa from the bases of the villi and into the lamina propria are called crypts of Lieberkühn, more prominent in the proximal small intestine compared to the distal part (Helander and Fändriks 2014, Parker and Hohenberger 2019, Agarwal et al. 2021), acting as glands that secrete antimicrobial agents and hormones. To this scope, Paneth cells, highly specialized secretory cells, are located in the crypts. They contain prominent eosinophilic granules in their cytoplasm (Lueschow and McElroy 2020), composed of antimicrobial peptides and immunomodulating proteins, that once released at the apical surface into the lumen, regulate the composition and abundance of the intestinal microbiota and protect from pathogens (Lueschow and McElroy 2020). Lastly, specialized microfold cells (M cells) cover organized lymphoid follicles in the ileum, called Peyer’s patches. They play a central role in initiating mucosal immune response by transport antigens and microorganisms to the underlying lymphoid tissue. In fact, ablation of M cells in mice results in delayed maturation of Peyer’s patches and inefficient induction of secretory IgA (Rios et al. 2016). In addition to these protective barriers, a physical barrier is guaranteed by the presence of cell–cell junction complexes. These protein complexes are involved in cell–cell adhesion, preventing paracellular diffusion of microorganisms and antigens while regulating paracellular transport of molecules. Junctional complexes include tight junctions, the most apical component of intracellular junctions.

To support the protection and digestive function of the epithelium, submucosal Brunner’s glands are located in the first and distal portion of the duodenum, and secrete several products, such as a bicarbonate-rich alkaline secretion to neutralize the acid chyme, a mucinous secretion, to lubricate the mucosa, bactericidal factors, epidermal growth factor, and surface-active lipids (Gelberg 2014, Bass and Wershil 2015). Moreover, by conveying a rich network of blood vessels, lymphatics, and nerves, the submucosa supports the mucosa in nutrient, fluid, and electrolyte absorption.

The absorptive and protective functions of the gut are dependent on an intact and functional epithelium, maintained by constant cell renewal. In adult mammals, the intestinal epithelium undergoes continuous turnover every 2–5 days (Darwich et al. 2014) from the pool of multipotent stem cells, residing at the base of the small intestinal crypts. These cells have been well-characterized and are known to express stem cell markers such as a Leu-rich repeat-containing G-protein-coupled receptor (LGR5) (Barker et al. 2007), fundamental for intestinal homeostasis (Tan et al. 2021). In fact, the mature cell type that constitutes the epithelium, originates from stem cells and differentiate during the migration away from the replicative zone at the bottom of the crypt, along the crypt–villus axis (Barker 2014, Agarwal et al. 2021). Active intestinal stem cells, also known as crypt base columnar cells (CBCs), spaced alternatively to Paneth cells, undergo constant proliferation, and give rise to transit-amplifying cells. These cells differentiate into absorptive lineage, giving rise to mature enterocytes and secretory lineages, from which goblet cells, EECs, Tuft cells, and M cells mature. To guarantee the preservation of the epithelium, both proliferative progenitors and terminally differentiated cells can ‘revert’ to an intestinal stem cell phenotype, following depletion of the Lgr5 + population, to support tissue regeneration (Tetteh et al. 2016). Additionally, a quiescent stem cell population commonly referred to as + 4 label retaining cells (+4 LRC), is also present in the crypt and is able to restore the LGR5 + CBC stem cells, when depleted (Tian et al. 2011).

## The small intestinal microbiota

In the small intestine, the main phyla described are Firmicutes, Proteobacteria, Bacteroidetes, Fusobacteria, and Actinobacteria, recently renamed as Bacillota, Pseudomonadota, Bacteroidota, Fusobacteriota, and Actinomycetota, respectively. However, along the small intestinal segments, differences exist in bacterial composition and abundance. The small intestinal microbiota can be considered an open ecosystem receiving an influx of microorganisms from proximal locations of the digestive tract. An important determinant of the small intestinal microbiota composition is the oral cavity. Daily, about 1–1.5 l of saliva is swallowed, (Humphrey and Williamson 2001) resulting in the ingestion of about 10^12^ bacteria per day. When ingested, these oral bacteria need to conquer multiple chemical and physical barriers, gastric acid, and bile acids before colonizing further along the gastrointestinal tract (Martinsen et al. 2005, di Gregorio et al. 2021). In healthy individuals, 89% of the taxa present in the duodenum are also found in paired saliva samples (Barlow et al. 2021), indicating a huge impact of the oral–intestinal transfer in the determination of microbial composition. Likewise, the microbial community from the jejunum resembles that of the duodenum microbial community (Nagasue et al. 2022), and hence also overlaps with the oral community, including *Prevotella, Veillonella, Haemophilus*, and *Fusobacterium* (Sundin et al. 2017). Conversely, the ileum, which shows significant differences from the jejunum composition, clusters between the upper and lower gastrointestinal tract (Nagasue et al. 2022). The transfer of oral-like bacteria to the ileum is a certainty as even comparison between saliva and stool, shows oral–fecal transmission for members of oral *Streptococcus, Veillonella, Actinomyces*, and *Haemophilus*, while members of the *Prevotella* genus are only occasionally transmitted (Schmidt et al. 2019). When comparing the bacterial α-diversity of the upper, lower intestinal tract and fecal samples, the small intestine is reported to have the lowest α-diversity (Seekatz et al. 2019, Vuik et al. 2019, Kashiwagi et al. 2020). Additionally, when compared with saliva, the jejunum was also found to have a lower diversity than saliva (Sundin et al. 2017), possibly due to the drastic bacterial reduction in the stomach. In this section, the bacterial community in the adult small intestinal segments (duodenum, jejunum, and ileum), is described.

### Duodenum

The duodenal microbial load in a healthy individual is considered lower than or equal to 10^3^ CFU/ml of duodenal aspirate, which is also the threshold to define a disease condition termed SIBO (detailed in a dedicated section) (Pimentel et al. 2020). The overall duodenum luminal (aspirates) and mucosal (biopsies) bacterial community is dominated by Bacillota and Pseudomonadota accounting together for more than 70%, while Bacteroidota, Actinomycetota, and Fusobacteriota are present at lower levels (Li et al. 2015, Vuik et al. 2019, Leite et al. 2020b, Nagasue et al. 2022). Leite and colleagues identified Actinomycetota as second dominant phyla after Bacillota and in other studies some individuals present high levels of Bacteroidota, making Bacillota, Pseudomonadota, Actinomycetota, and Bacteroidota the dominant phyla in the duodenum, followed by Fusobacteriota and TM7 (Li et al. 2015, Seekatz et al. 2019, Kashiwagi et al. 2020, Leite et al. 2020b). At genus level, most studies report *Streptococcus* (*Streptococcaceae*) as one of the dominant bacteria in the duodenal lumen and mucus (Li et al. 2015, Seekatz et al. 2019, Vuik et al. 2019, Kashiwagi et al. 2020, Barlow et al. 2021, Nagasue et al. 2022). Other occurring genera, reported in duodenum in healthy conditions, are summarized in Table 4.

**Table 4.** Bacterial genera described in the human small intestine luminal and mucosal regions. The table includes the top five genera (or higher level if not described down to genus level) detected in each study.

	Bacillota	Pseudomonadota	Bacteroidota	Actinomycetota	Fusobacteriota	Other
***Duodenum***						
Lumen	*Carnobacteriaceae* ^3b^, *Gemella*^2^, *Gemellaceae*^3b^, *Lactobacillaceae*^3b^, *Streptococcaceae*^3b^, *Streptococcus*^1,2,3a^, *Veillonellaceae*^3b^, *Veillonella*^2^,^3a^	*Enterobacteriaceae* ^1,3a^, *Escherichia–Shigella*^1^, *Haemophilus*^1^, *Pasteurellaceae^2^*	*Prevotella* ^2^	*Rothia* ^3a^	*Fusobacterium* ^1,3a^	
Mucosa	*Faecalibacterium* ^6^, *Lactobacillus*^6b^, *Streptococcaceae*^4^, *Streptococcus*^5,6a^, *Veillonellaceae*^4^, *Veillonella*^5^	*Acinetobacter* ^6b^, *Bradyrhizobiaceae*^4^, *Escherichia*^5^, *Haemophilus*^5^, *Pseudomonadaceae*^4^, *Stenotrophomonas*^6a^	*Bacteroides* ^6^, *Prevotellaceae*^4^, *Prevotella*^5,6^			
***Jejunum***						
Lumen	*Carnobacteriaceae* ^3b^, *Clostridiaceae^3b^*, *Gemella*^2a,2c^, *Lactobacillaceae*^2,3b^, *Streptococcaceae*^3b^, *Streptococcus^2^*,^7^,^3a^, *Veillonellaceae*^3b^, *Veillonella*^2^,^7^,^3a^	*Enterobacteriaceae* ^2b,2c,3a^, *Escherichia*^7^, *Pasteurellaceae*^2a,2b^, *Pseudomonas*^3a^	*Prevotella* ^7^	*Rothia* ^3a^	*Fusobacterium* ^7^	
Mucosa	*Clostridium IX* ^9^, *Clostridium XI*^9^, *Lactobacilli*^10^, *Streptococcaceae*^4^, *Streptococci*^10^, *Streptococcus*^5,8,9^ , *Veillonellaceae*^4^, *Veillonella*^5,8^	*Actinobacillus* ^10^, *Bradyrhizobiaceae*^4^, *Enterococcus*^10^, *Escherichia*^5^, *Haemophilus*^5,8^, *Klebsiella*^10^, *Pseudomonadaceae*^4^, *Proteobacteria*^9^	*Bacteroidetes* ^9^, *Prevotellaceae*^4^, *Prevotella*^8^	*Actinomyces* ^5^, *Rothia*^8^	*Fusobacteria* ^9^	
***Proximal Ileum***						
Lumen*	*Clostridium I* ^11^, *Streptococcus*^11^, *Veillonella*^11^	*Enterococcus* ^11^, *Oxalobacter*^11^				
Mucosa	*Streptococcaceae^4^, Streptococcus* ^5^, *Veillonellaceae*^4^, *Veillonella*^5^	*Comamonadaceae* ^4^, *Escherichia*^5^, *Haemophilus^5^, Pseudomonadaceae*^4^	*Bacteroides* ^5^	*Micrococcaceae* ^4^		
***Terminal ileum***						
Lumen	(no data)					
Mucosa	*Clostridium XIVa^5,9^*, *Clostridium IV^9^*, *Clostridium IX*^9^, *Clostridium XIVb^9^*, *Granulicatella*^13^, *Lachnospiraceae*^4^, *Ruminocacceae*^4^, *Streptococcus*^5,13^, *Veillonellaceae*^4^	*Acinetobacter* ^12^, *Aeromonadaceae*^12^, *Cupriavidus*^12^, *Enterobacteriaceae*^4,12^, *Escherichia*^5^	*Bacteroidaceae* ^4^, *Bacteroidetes*^9^, *Bacteroides*^5^	*Actinomyces* ^13^, *Rothia*^13^	*Cetobacterium* ^12^, *Fusobacterium*^5^	*Verrucomicrobiaceae* ^9^, *TM7(G-1)*^13^

References:

^1^Barlow et al. (2021), ^2a^Seekatz et al. (2019) (proximal jejunum), ^2b^Seekatz et al. (2019) (mid jejunum), ^2c^Seekatz et al. (2019) (distal jejunum), ^3a^Leite et al. (2020b) (group 2), ^3b^Leite et al. (2020b) (group 3), ^4^Vuik et al. (2019), ^5^Nagasue et al. (2022), ^6a^Li et al. (2015) (Mucus – luminal fluid), ^6b^Li et al. (2015) (Mucosal biopsies); ^7^Sundin et al. (2017); ^8^Dlugosz et al. (2014), ^9^Wang et al. (2005), ^10^Hayashi et al. (2005), ^11^Booijink et al. (2010), ^12^Fan et al. (2020), and ^13^Villmones et al. (2018).

*(Based on ileostomy samples)

In a study by Li et al. (2015), the microbial composition of duodenal biopsies and duodenal fluid was compared, and they observed dominant microbes differing between both samples. In particular, while the biopsies were dominated by *Acinetobacter, Bacteroides*, and *Prevotella*, in the duodenal fluid *Prevotella, Stenotrophomonas*, and *Streptococcus* were abundant. Yet, reports comparing the microbial composition in the mucosal and luminal niche are limited and additional research is needed to fully appreciate how this niche-specific community varies in the human duodenum, not only at interindividual, but also at intraindividual level.

### Jejunum

The jejunum load ranges from 5.8 × 10^3^ to 8.0 × 10^6^ CFU/ml when sampled during enteroscopy (Sundin et al. 2017), yet when sampled during surgery, lower bacterial levels < 1.6 × 10^3^ were detected in the median population (Villmones et al. 2022). In terms of oxygen resistance, the jejunal luminal microbiota are primarily aerobes, facultative and obligate anaerobes and oxygen-tolerant bacteria (Hayashi et al. 2005, Sundin et al. 2017). The jejunal lumen and mucosa are dominated by Bacillota and Pseudomonadota, followed by Bacteroidota, Actinomycetota, and Fusobacteriota in varying levels, depending on the study (Wang et al. 2005, Dlugosz et al. 2014, Sundin et al. 2017, Vuik et al. 2019, Leite et al. 2020b). In comparison to the duodenum, jejunal biopsies and aspirates present lower levels of Bacteroidota, among which the genus *Prevotella* (Seekatz et al. 2019, Leite et al. 2020b, Nagasue et al. 2022). While *Prevotella* was reported within the top three most abundant genera in the jejunal mucosa and lumen of healthy individuals, no comparison to duodenum samples was made in these studies (Dlugosz et al. 2014, Sundin et al. 2017).

Similar to the duodenum, the jejunum at genus level is dominated by *Streptococcus* in most studies (Hayashi et al. 2005, Dlugosz et al. 2014, Sundin et al. 2017, Vuik et al. 2019, Villmones et al. 2022), while the presence of other genera greatly differs between reports (Table 4). A study by Sundin et al. (2017) on jejunal aspirates, identified six core species, defined as abundant in more than 50% of the subjects, namely: *Streptococcus mitis, Veillonella atypica, Haemophilus parainfluenzae, Fusobacterium periodonticum, Streptococcus vestibularis*, and *Prevotella melaninogenica*. Dlugosz et al. (2014) observed a clustering of about 24% of the jejunal mucosal samples dominated by *Prevotella*, the remaining samples were distributed along a gradient between a high *Streptococcus* or *Escherichia* abundance. In addition, they reported patterns of codependence between *Prevotella* and *Veillonella* and mutual exclusivity between *Escherichia* and *Rothia*.

### Ileum

When describing the ileum microbiota, a distinction is made between the proximal and terminal part, usually sampled in a different manner, which possibly introduces cross-contaminations from upper or lower gastrointestinal tract, respectively. The proximal ileum mucosa is dominated by Bacillota and Pseudomonadota (Vuik et al. 2019, Nagasue et al. 2022), while the terminal ileum mucosa has increased Bacteroidota levels compared to the proximal ileum (Wang et al. 2005, Vuik et al. 2019, Nagasue et al. 2022). However, when sampling the distal ileum (and sometimes the proximal too) a retrograde endoscopy method is used with possible cross-contamination from the lower gastrointestinal tract, which harbours increased Bacteroidota levels. To limit the cross-contamination, Villmones et al. (2018) sampled the terminal ileum directly during surgery and reported that Bacillota was predominant, followed by Actinomycetota, Candidate division TM7, Pseudomonadota, and Fusobacteriota while Bacteroidota was only found in 40% of the subjects. However, conflicting results are described in a study on terminal ileum biopsies, where Fusobacteriota dominates, followed by Pseudomonadota, Bacillota, Bacteroidota, and Actinomycetota (Fan et al. 2020). These contradictory results might be due to the different sampling method, the demographic parameter of the studied population (e.g. age) and other possible confounders, such as diet and pathologies (Booijink et al. 2010, Barlow et al. 2021, Leite et al. 2021). In contrast to the upper small intestinal sites, the Verrucomicrobia phylum seems to primarily appear in the terminal ileum (Wang et al. 2005, Nagasue et al. 2022).

At genus level, the ileum displays increased *Bacteroides* and *Escherichia* levels, but lower *Prevotella* levels, compared to the duodenum and jejunum (Nagasue et al. 2022).

As for the jejunum, a core microbial ileal community was described (genera present in > 50% of samples) in terminal ileum mucosa, composed of *Streptococcus, Actinomyces, Gemella, Rothia, Oribacterium, TM7 (G-1), Fusobacterium, Granulicatella, Bifidobacterium, Solobacterium, Eubacterium, Atopobium, Lachnoanaerob, Parvimonas, Stomatobactulum*, and *Abiotrophia* (Villmones et al. 2018). Additionally, a core of eight phylogenetically related groups, common in four ileostomy effluents, was defined by species belonging to *Veillonella, Streptococcus, Clostridium* cluster I, and *Enterococcus* (Booijink et al. 2010). Both studies present a different core, yet they also sampled a different location. Indeed, while ileostomy effluent has been shown to cluster closely to jejunal samples, ileum samples from healthy adults positioned between ileostomy effluent and fecal samples (Zoetendal et al. 2012).

In terms of diversity, ileum biopsies were found more diverse than jejunal biopsies (Nagasue et al. 2022). Indeed, intraindividual differences in ileum effluent are described to be, overall, higher than in fecal samples, and show daily fluctuations, possibly impacted by diet or other confounders. Over a period of 9 days, about 44% similarity was observed in ileostomy effluent, while fecal samples are found to have about 92% similarity over a period of minimal 2 months (Rajilić-Stojanović et al. 2009, Booijink et al. 2010). The *Streptococcus* population shows high diversity in ileostomy effluent as seven genetic lineages (not all within one sample) were observed closely related to *S. salivarius, S. thermophilus* (*S. salivarius* species group), and *S. parasanguinis* (*S. mitis* species group). In contrast, the *Veillonella* genus represents less diversity, as all belonged to the same genetic lineages (Van den Bogert et al. 2013).

### Host–bacterial–diet interaction in nutrient digestion and absorption

The complex microbial community residing in the small intestine encompasses diverse metabolic activities, pivotal for the digestion of nutrients, otherwise not accessible for host absorption. In this section, we describe how the host digestive processes are supported by microbial metabolic functions for the digestion of carbohydrates, proteins, lipids, and some micronutrients, hence contributing to nutrient absorption.

The distal small intestinal microbiota undergoes the hydrolysis of nondigestible carbohydrates, resistant to host’s digestive enzymes, through carbohydrate-active enzymes (CAZymes), with SCFAs as end-products. To date, in the human gut microbiome, 15 882 different CAZyme genes have been identified (Kaoutari et al. 2013), classified based on amino-acid sequence similarities, into five families: (i) glycoside hydrolases (GHs), the most prevalent among the gut bacteria, responsible for the hydrolysis and/or transglycosilation of the glyosidic bonds; (ii) glycotransferases (GTs), catalyzing the glycosidic bond formation by transferring a moiety from an activated donor molecule to specific donor molecules; (iii) polysaccharide lyases (PLs), a group of 31 enzymes which cleave uronic acid-containing polysaccharide chains; (iv) carbohydrate esterases (CEs), which remove ester-based modification in mono-, oligo- and polysaccharides, hence facilitating the action of GHs on complex polysaccharides; and (v) carbohydrate-binding modules (CBMs), often associated to other CAZyme and without enzymatic activity *per se*, are dedicated to facilitate the enzyme–substrate interaction and potentiate the enzymatic activity (Davies et al. 2005, Cantarel et al. 2009) (http://www.cazy.org), extensively reviewed in Wardman et al. (2022).

By functional metagenomic screening of a fosmidic library constructed from ileal mucosa, Patrascu et al. (2017) demonstrated that the ecosystem in the human ileal mucosa, harbours the fibrolytic potential to catabolize complex and diversified plant cell wall polysaccharides. In particular, they identified 25 enzymes dedicated to carbohydrate metabolism (21 GH, 2 CE, and 1 GT) from *Bacteroides* and *Eubacterium* related species, mainly responsible for plant-cell-wall degradation, but also starch and fructose-based saccharide degradation in the ileum (Patrascu et al. 2017). Moreover, by comparative functional analysis, several pathways and functions for carbohydrate uptake and metabolism are described as enriched in the small intestinal metagenome, compared with those of fecal metagenomes, suggesting that uptake and fermentation of available carbohydrates in the small intestinal lumen contributes to the maintenance of the resident microbiota. In particular, genes involved in the expression of several sugar phosphotransferase systems (PTS), enzymes related to central metabolism (e.g. pentose phosphate pathway), and fermentation pathways (e.g. lactate and propionate fermentation) are highly enriched in the small intestinal microbiome (Zoetendal et al. 2012). Zoetendal et al. (2012) also reported that genes linked with PTS transcription are mainly expressed by streptococci, suggesting that these bacteria are the main utilizers of available carbohydrates in the small intestinal lumen. Coherently, genes assigned to the butyrate fermentation pathway are reported in the human small intestinal microbiome (Zoetendal et al. 2012), although limited compared to the enrichment described in the metagenome of the large intestine, which is in line with the higher abundance of butyrate producers in colon (Pryde et al. 2002).

The microbial utilization of amino acids starts in the small intestine and the bacterial composition associated with protein metabolism has been described. Indeed, a shift in ileal microbiota composition is reported in response to the intake percentage (Qiu et al. 2018) and sources (Kar et al. 2017) of dietary proteins, in animal models. In particular, bacterial richness and SCFAs concentration in the ileum decrease with the reduction of protein intake (Qiu et al. 2018). Additionally, the proportion of *Clostridium_sensu_stricto* and *Escherichia–Shigella* decreases and increases, respectively, with the reduction of protein intake (Qiu et al. 2018). As well, the source of protein (peptides or amino acids) impact the *Lactobacillus* colonization dominance in the pig small intestine, leading to the prevalence of *Lactobacillus amylovorus* in peptide-rich environment (Jing et al. 2022). Furthermore, through culturing approaches, it was reported that *Klebsiella* spp., *Streptococcus* spp., *E. coli*, and *Mitsuokella* spp. from the porcine small intestine utilize amino acids at a rate of 50%–90% over 24 h, suggesting a potentially relevant impact on the overall small intestinal ecology (Dai et al. 2010). Although similar studies on differential protein intake have not yet been conducted on humans, to our knowledge, these results suggest an important role of small intestinal microbiota in protein utilization. Indeed, in human ileal aspirates, enzymes related to amino acid metabolism are highly enriched, compared to fecal samples (Zoetendal et al. 2012). It is possible that, considering the rapid host uptake of peptides and amino acids present in the small intestinal lumen, the *de novo* synthesis of amino acid by the small intestinal microbiome is stimulated (Zoetendal et al. 2012).

The lipid metabolism in the human gut is indirectly linked to the small intestinal microbiota activity, that convert conjugated primary bile acids from the host into deconjugated analogues and subsequently convert the primary into secondary bile acids. The deconjugation of primary bile acid reaction is catalyzed by the activity of the BSH.

From a construct metagenomic dataset of sequences from different cohorts worldwide, Song and colleagues reported that BSH sequences are distributed in 591 intestinal bacterial strains (Song et al. 2019). Indeed, BSH activity provide an ecological advantage by enhancing the resistance to the conjugated bile acids and promotes the survival and colonization in the intestine (Jones et al. 2008). Those unconjugated primary bile acids are converted into secondary bile acids following a C-7-epimerization and a 7-alpha-dehydroxylation, encoded by the bile acid-inducible (bai) *baiB* gene (Ye et al. 1999) by *Bacteroides, Eubacterium*, and *Clostridium* genera. Within the Actinomycetota , *Bifidobacterium* species possess two major BSH enzyme types: A and C with a highest specificity for the glycine-conjugated bile salts over taurine-conjugated forms (Kim et al. 2004).

Upon lipid digestion, fatty acids are taken up by enterocytes *via* both protein-mediated and protein-independent transport.

Along with their role in the digestion of carbohydrates, proteins, and bile salts metabolism, intestinal microbiota can synthesize certain vitamins, notably B group vitamins and vitamin K (Hill 1997). These vitamins, important for bacterial metabolisms, also have a metabolic and physiological significance in humans. In fact, humans exposed to low vitamin K diet during 3–4 weeks did not develop vitamin deficiency, in contrast to those administered with a large spectrum antibiotic (Frick et al. 1967). However, the majority of the studies focus on the overall gut and fecal microbial community and, to our knowledge, no report specifically focused on bacteria isolated from small intestine. Nonetheless, by genome annotation of 256 human gut bacteria, the biosynthesis pathways for eight B-vitamins (B8, B12, B9, B3, B5, B7, B2, and B1) was predicted in 40%–65% of the analyzed genomes and the majority of these predictions matched published experimental data (Magnúsdóttir et al. 2015).

Animals are incapable of synthesizing cobalamin (vitamin B12), and thus rely on dietary sources of cobalamin. In humans, cobalamin uptake takes place in the ileum. As such, microbial vitamin B12 produced further along the intestine is not absorbed by the host but, instead, used to synthesize other corrinoids, not used by the human. The bacterial synthesis of cobalamin can be performed either aerobically or anaerobically but human gut microbiota preferentially uses the anaerobic route (Magnúsdóttir et al. 2015). It has been shown that cobalamin biosynthetic pathways involve nearly 30 different enzymes, including *hemBCD, cbi*, and *cob* genes (Taranto et al. 2003, Piwowarek et al. 2018). Within the human gut microbiome, the synthesis of cobalamin was predicted in most of Fusobacteriota, rare in Actinomycetota and Pseudomonadota while missing in half of the genomes in the Bacteroidota and Bacillota phyla (Magnúsdóttir et al. 2015). Besides bacterial vitamin B12 production, some bacteria also utilize vitamin B12, essential for enzyme cofactors and gene regulations (Degnan et al. 2014, Wexler et al. 2018).

Folate (vitamin B9) in the gut, involved in major metabolic pathways such as amino acid conversion and nucleotide synthesis, mostly derives from two sources: one from the dietary products, which is absorbed by the small intestine enterocytes, and another, which is the by-product of dietary fibers fermentation by the gut microbiota and, is absorbed then in the colon. *De novo* folate biosynthesis involves both 6-hydroxymethyl-7,8-dihydropterin pyrophosphate (DHPPP) and para-aminobenzoic acid (pABA) as precursors (Rossi et al. 2011). By systematic analysis of the overall human microbiota genome, it is reported that folate biosynthesis pathway, while rare in Actinomycetota and Bacillota genomes, is present in almost all Bacteroidota, Fusobacteriota, and Pseudomonadota genomes (Magnúsdóttir et al. 2015). This suggest a relevant impact on host’s folate metabolism, although report focusing specifically on human small intestinal microbiome and folate synthesis are not yet available. Lastly, biotin (vitamin B7) is required for normal cellular function and development, yet humans and other mammals are not able to synthesize it. Exogenous biotin needed to satisfy the nutritional requirement is provided through two sources: the diet and the microbial production. In the human small intestinal microbiome, biotin synthesis genes are described, phylogenetically linked primarily to Pseudomonadota but also associated with Bacillota and Bacteroidota (Zoetendal et al. 2012). Moreover, since biotin absorption by epithelia takes place in the intestine (Said 2009), it is plausible that small intestinal bacteria may contribute to the host’s biotin supply (Zoetendal et al. 2012).

## Disruption of small intestinal homeostasis in nutrient balance

It is evident that the small intestine is pivotal in nutrient digestion and absorption. As such, any disruption of its homeostasis can lead to an altered microbial ecology and metabolic activity toward the dietary component present in the lumen. Consequently, the altered small intestinal environment may compromise nutrient absorption and ultimately result in malnutrition. In this context, an expanded knowledge on small intestinal ecology in healthy and disease conditions is crucial to define or adapt therapeutic approaches to improve nutritional status. The conditions impacting small intestinal homeostasis and subsequent nutritional status can be diverse, ranging from microbial dysbiosis and pathogen infections to inflammatory diseases and anatomic changes. In the next section we will describe two small intestinal conditions, namely short bowel syndrome (SBS) and small intestinal bacterial overgrowth (SIBO), as examples to highlight the drastic impact from physical resection or bacterial dysbiosis on host nutrient status.

### Short Bowel Syndrome

Short Bowel Syndrome (SBS) is a rare and severe condition defined by an extensive loss of small intestinal surface. The most frequent causes of SBS, in adults, are mesenteric ischemia, Crohn’s disease, radiation enteritis, postsurgical intra-abdominal adhesions, and postoperative complications (Pironi et al. 2006). In adults, where normal small intestinal length is approximately 600 cm, SBS is defined by a remaining small bowel in continuity of less than 200 cm and it is classified based on anatomical, pathophysiological, and postoperative evolution criteria (Pironi et al. 2015). According to anatomical criteria, three types of SBS are defined: (i) type I, end-jejunostomy with no colon in continuity; (ii) type II, jejuno–colic anastomosis, where the remnant jejunum is in continuity with part of the colon; and (iii) type III, jejuno–ileal anastomosis with ileo–cecal valve and the intact colon in continuity (Jeppesen 2014) (Table 5). The extensive removal of small intestinal surface results in intestinal failure, defined as the insufficient digestion of nutrients and hence requiring parenteral nutrition to sustain the metabolism and prevent malnutrition and dehydration (Pironi et al. 2015). The real incidence and prevalence of SBS is unclear, due to the lack of reliable patient databases but, based only on the patients receiving home parenteral nutrition (5–80 per million population in Europe), SBS is estimated to contribute for 75% of them (O’Keefe et al. 2006, Pironi et al. 2006, Jeppesen 2014).

**Table 5.** Anatomical classification of SBS and its characteristics.

	Type I SBS	Type II SBS	Type III SBS
Surgical procedure	End-jejunostomy	Jejuno–colic anastomosis	Jejuno–ileal anastomosis
	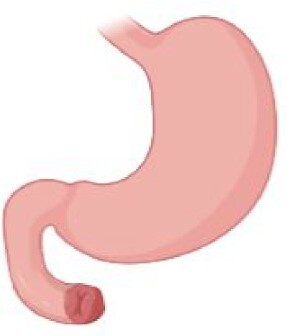	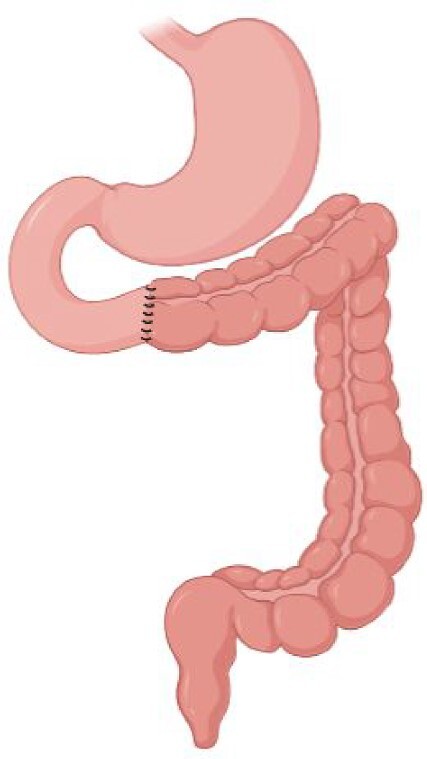	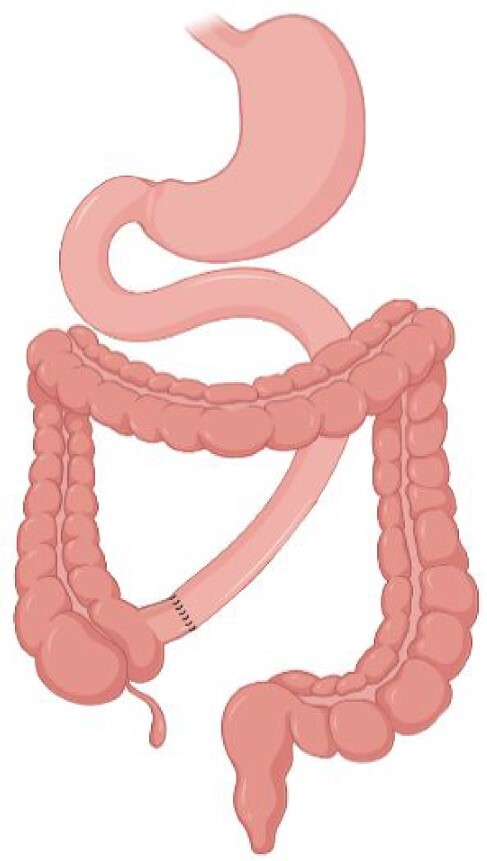
**Preservation of ileocecal valve**	No	No	Yes
**Preservation of colon**	No	Partial	Complete

After surgery and first acute intestinal failure stage, a spontaneous process termed adaptation follows, characterized by morphological, histological, and metabolic changes in the intestinal mucosa to compensate for the reduced absorptive area (Jeppesen 2014, Klek et al. 2016). The clinical and metabolic conditions of patients in a state of chronic SBS strongly depend on the intestinal segment affected, the functionality of the remaining intestine and the presence or absence of terminal ileum, ileocecal valve, and colon (Pironi et al. 2016). Yet, the adaptive responses as well as the factors involved in this interindividual variation are still poorly characterized and only approximately half of the patients can achieve nutritional autonomy (Amiot et al. 2013). This suggests that interindividual variations exist and may determine SBS evolution (Mayeur et al. 2016). Based on morphological and histological alterations, adaptation is characterized by intestinal dilatation, increased area and length of the villi, expanded number of goblet cells, and elevated intestinal epithelial sodium permeability (reviewed in Billiauws et al. 2018). The most notable response occurs in the residual ileum, with increased enterocyte proliferation as soon as 8 h after resection in a SBS animal model (Dahly et al. 2003), indicating the importance of preserving residual ileum and colon in continuity. When the distal ileum and colon are removed, a loss of gastric emptying inhibition (ileal brake) is observed immediately after the intestinal resection, because of the extensive anatomical alteration and the crucial contribution of the small intestine in gastric emptying (through the production of peptide hormones ghrelin and GLP-1), resulting in fast transit of food, gastric acid, and bile acid (Nightingale et al. 1993). Conversely, in patients with colon in continuity, gastric emptying and transit time are comparable with nonresected condition (Nightingale et al. 1993).

Such alterations in transit, along with the increase in oxygen level, gastric and bile acids concentrations in the remnant bowel, strongly influence the intestinal ecology, and result in a significant decrease in OTU counts and α-diversity and, overall, a diverse microbial community structure between healthy and SBS conditions and among different SBS types (Huang et al. 2017, 2020, Zeichner et al. 2019, Hu et al. 2021) (Summarized in Table 6). Interestingly, a greater proportional abundance of fecal *Enterobacteriaceae* and lactobacilli were correlated with a longer and shorter parenteral nutrition duration, respectively (Huang et al. 2017), suggesting that the intestinal ecology upon extensive small bowel resection may contribute to the evolution of SBS and the achievement of nutritional autonomy. Coherently, type III SBS patients, for whom ileocecal valve and colon are preserved, have relatively quicker intestinal adaptation and better clinical outcome. Nevertheless, overload of *Lactobacillus* in SBS can also be deleterious, in preventing implantation of other bacteria such as *Clostridium* clusters (Joly et al. 2010). It is noteworthy that the roles of *Lactobacillus* in SBS patients are complicated and variable. While lactobacilli abundance is associated with shorter parenteral nutrition duration in human (Huang et al. 2017), the capacity of certain lactobacilli to produce *D*-lactic acid, coupled with the reduced absorption potential, exposes SBS patients to a high risk of *D*-lactic acidosis (Mayeur et al. 2013) and neurological disorders (Mayeur et al. 2013, Kowlgi and Chhabra 2015).

**Table 6.** Reported taxonomical changes (phylum and genus level) in SBS patients or animal models.

				Phylum		Genera		
Human/animal (number)	SBS type	Surgical procedure	Control condition	Relative increase in SBS	Relative decrease in SBS	Relative increase in SBS	Relative decrease in SBS	References
Human (five/group)								
	II	Jejuno–colonic anastomosis	Healthy control	Pseudomonadota	Bacillota Bacteroidota	*Proteus* *Klebsiella* *Streptococcus Megasphaera*	*Blautia* *Dorea* *Lachnospira Anaerostipes Fusicatenibacter Roseburia Pseudobutyrivibrio Flavonifractor* *Faecalibacterium Bacteroides*	Huang et al. (2017)
	III	Jejuno–ileal anastomosis	Healthy control	Bacteroidota	–	*Lactobacillus Prevotella*	*Blautia* *Dorea* *Lachnospira Anaerostipes Fusicatenibacter Roseburia Pseudobutyrivibrio Flavonifractor Faecalibacterium Bacteroides*	
Human (11 patients, eight controls)	II	Jejuno–ileal anastomosis	Colonoscopy patients	–	–	*Lactobacillus*	*Clostridium spp*.	Joly et al. (2010)
Rat (five/group)	II	Ileocecum resection and jejuno-colostomy	Transection and reanastomosis without small bowel removal	Bacillota Pseudomonadota Actinomycetota	Bacteroidota Verrucomicrobia Tenerictes Deferrribates TM7 Unclassified phyla	*Proteus*	*Bacteroides Odoribacter Prevotella Coprococcus* *Dorea* *Oscillospira Ruminococcus Clostridium Akkermansia*	Hu et al. (2021)
Rat (10/group)								
	II	75% small intestinal resection, ileo–cecal junction resected	Transection and reanastomosis without small bowel removal	Pseudomonadota Fusobacteriota	Bacteroidota Actinomycetota Verrucomycrobia	*Lactobacillus Escherichia–Shigella* *Klebsiella* *Proteus Pasteurella*	*Akkermansia* *Blautia Butyrivibrio Lachnoclostridium Roseburia Anaerotruncus Butyricicoccus Flavonifractor Ruminiclostridium Allobaculum Desulfovibrio*	Huang et al. (2020)
	III	75% small bowel resection, ileo–cecal junction preserved	Transection and reanastomosis without small bowel removal	Fusobacteriota	Bacillota, Veruucomicrobia	–	–	

In SBS patients, the shifted fecal microbial ecology is reflected in altered functional profiles of carbohydrate and amino acid metabolisms, along with the depletion in anaerobic Bacillota and prevalence of facultative anaerobic Pseudomonadota (Huang et al. 2017). Coherently, dietary supplementation with the pyrimidine precursor orotate and uracil stimulated jejunal adaptive growth in a SBS animal model (Evans et al. 2005). As well, other microbial metabolic pathways reduced in SBS patients include methane metabolism and oxidative phosphorylation, suggesting insufficient energy harvest (Huang et al. 2017).

Furthermore, the changes in the intestinal environment after intestinal resection expose SBS patients to the development of SIBO, a condition characterized by excessive number of bacteria in the small intestine (further detailed in the next section).

Overall, studies on SBS animal models and patients indicated that achieving intestinal adaptation and nutritional autonomy is not only influenced by the extensiveness of the removed small intestine (Berlin et al. 2019, Sun et al. 2020) but also, by the remaining colon and early plasma citrulline concentrations (Amiot et al. 2013). However, considering the evidences on the role of small intestinal microbiota in nutrient digestion and absorption, we cannot exclude that the ecological alterations in the remnant intestine have an impact also on host’s adaptation, although not yet elucidated, to our knowledge. Coherently, parenteral nutrition supplemented with SCFAs and, in particular, butyrate at physiological concentrations, is described to improve structural and metabolic adaptation (i.e. increased villus height, number of crypt cells and plasma GLP-2 concentrations) in the small intestine, accelerates adaptation and potentially shortens the period of full adaptation, in a SBS piglet model (Jeppesen et al. 2000, Bartholome et al. 2004, Filippi et al. 2021). Furthermore, after reinfusing proximal jejunostomy output into the distal part of the small intestine Liu et al. (2016) observed a shorter parenteral nutrition period, protected integrity of the intestinal mucosa and increased nutrient absorption, in particular when the ileocecal valve was preserved. Although the mechanisms for this amelioration were not fully elucidated, it is plausible that by reinfusing jejunostomy output, also jejunum bacteria are added into the distal intestine, leading to a modulation of the overall ecology.

In terms of nutritional requirement of SBS patients, the gastrointestinal anatomy after resection is crucial to personalize the nutritional needs. For example, while jejuno–colonic anastomosis patients should receive about 30–35 kcal/kg/day of complex carbohydrates with soluble fibers, this is not necessary for patients without remnant colon, but relevant is the supplementation in long-chain triglycerides (Buchman et al. 2003). Along with ensuring the appropriate nutrient intake, the current therapeutic approach for SBS patients relies on antisecretory, antidiarrheal, and antimotility (e.g. loperamide) drugs and somatostatin to reduce intestinal loss (Vílchez-López et al. 2021), proton-pump inhibitors and hormonal therapies with GLP-2 analogues (i.e. teduglutide) (Vorre et al. 2022), to maximize absorption and effectively reduce parenteral nutrition requirements (Schwartz et al. 2016, Lam et al. 2018, Joly et al. 2020), although not universally effective (Billiauws and Joly 2019). In particular, besides the intestinotrophic effect on the intestinal epithelium, GLP-2 treatment is described to partially ameliorate also the intestinal bacterial dysbiosis of SBS rats by significantly downregulating the relative abundance of *Proteus* genus and increasing the relative abundance of *Clostridium* genus in SBS rats (Hu et al. 2021).

In this challenging context, it emerges the need for a personalized and multidisciplinary approach for SBS management. To this aim, one European (INTENS, ID 668294) and one US (Clinical trial ID NCT03530852) innovative projects are exploring alternative strategies to improve the quality of life of SBS patients by creating a functional small bowel from patients’ own cells for autologous transplants and designing a fat predigestion device, respectively. As such, this challenging and innovative approaches have the potentiality to drive the SBS management toward more patient-based therapies, in the near future.

### Small Intestinal Bacterial Overgrowth

Small Intestinal Bacterial Overgrowth (SIBO) manifests in the small intestine and, as the name indicates, is associated with an excessive number of bacteria in the small intestine causing gastrointestinal complaints (Pimentel et al. 2020). Despite the fact that natural microbial counts in the duodenal–jejunal area have been reported to be approximately 10^3^ CFU/ml, the cut-off to define SIBO differs between studies. In the past, SIBO was defined by a microbial load ≥ 10^5^ CFU/ml, but recent validations based on healthy controls urged to consider a lower cut-off for the diagnosis (Khoshini et al. 2008, Rezaie et al. 2017).

SIBO patients usually experience bloating, diarrhea, abdominal discomfort, and in more severe cases steatorrhea, weight loss, anemia, nutritional deficiencies (e.g. vitamin B12 deficiency), and/or mucosal inflammation (Lema et al. 2020, Pimentel et al. 2020, Quigley et al. 2020). Yet, no association was found between the overall increased microbial load and typical SIBO gastrointestinal symptoms, in a human SIBO cohort (Barlow et al. 2021).The prevalence of SIBO varies among reports between 2% and 22%, depending on the studied disease and the diagnostic method (Lakshmi et al. 2010, Fasano et al. 2013, Ierardi et al. 2016, Niu et al. 2016, Ricci et al. 2018, Wu et al. 2019, Kowalski and Mulak 2022).

Predisposing factors for SIBO development can be anatomical, pharmacological, or pathological abnormalities (i.e. intestinal stasis, decreased gastric acid production, pancreatic or biliary secretion deficiency, and an increased ileocecal valve reflux) or a malfunctioning gastrointestinal immune response resulting in a reduced microbial barrier or microbial clearance (Chander Roland et al. 2017, Quigley 2019). Additionally, aging, female gender, and proton pump inhibitors have also been proposed as predisposing factors, although not consistently (Dukowicz et al. 2007, Choung et al. 2011, Erdogan and Rao 2015, Su et al. 2018, Shin et al. 2019, Ghoshal et al. 2022). A complicating feature for the diagnosis is that these risk factors often appear in other diseases, making SIBO intertwined with other pathologies among which gastroenterological disorders (e.g. inflammatory bowel disease, irritable bowel syndrome, SBS, nonalcoholic fatty liver disease, cirrhosis, diabetes, and cystic fibrosis (Ierardi et al. 2016, Rafiei et al. 2018, Ricci et al. 2018, Fitriakusumah et al. 2019, Ghosh and Jesudian 2019, Wu et al. 2019, Phyo et al. 2021, Shah 2021, Feng and Li 2022, Ghoshal et al. 2022), and other nongastrointestinal (or indirectly linked) diseases such as Parkinson’s and Alzheimer’s disease (Kuang et al. 2021, Kowalski and Mulak 2022).

SIBO diagnosis is usually based on direct or indirect quantitative measurements, through aspirate culturing approach or breath tests, respectively. In clinical practice, the simplest and most widely used and available tests are hydrogen and methane breath tests, used as indirect read-outs for the microbial gas production in the small intestine, instead of in the colon under normal conditions. At histological level, no remarkable difference was observed in biopsies, besides a lower villous to crypt ratio (<3:1) in SIBO individuals compared to controls (Lappinga et al. 2010). Laboratory results can indicate SIBO by increased folate, due to increased bacterial synthesis, or decreased vitamin B12 levels. Vitamin B12 deficiency can be the result of increased bacterial consumption by the higher bacterial load, competitive binding with cobalamin-like bacterial metabolites or damage of binding sites (Quigley, Murray and Pimentel 2020).

Aside from quantitative diagnosis, qualitative approaches characterizing the small intestinal community in SIBO might improve SIBO management as gastrointestinal symptoms in SIBO were associated to a high load of so-called disruptor taxa but not to an overall high microbial load (Barlow et al. 2021). Instead, a high microbial load might be confounded by dietary habits. Indeed, in a small study cohort, nonsymptomatic healthy individuals consuming a high fiber diet positively correlated with SIBO, based on positive duodenal cultures, but only the switch to a low fiber high simple-sugar diet evoked gastrointestinal complaints (Saffouri et al. 2019).

To date, few studies characterized the small intestinal community in these patients and reported the associations of SIBO with changes in the small intestinal ecology (summarized in Table 7). By comparing duodenal aspirates of SIBO and non-SIBO individuals, Barlow et al. (2021) identified seven disruptor bacterial taxa, containing human pathogenic strains, that appear to displace common strict anaerobes. The taxa pointed out were *Enterobacteriaceae, Escherichia–Shigella, Clostridium sensu stricto_1, Enterococcus, Romboutsia, Aeromonas*, and *Bacteroides*. Among those, *Enterobacteriaceae* and *Escherichia–Shigella* were most commonly found in SIBO samples (Barlow et al. 2021) and, when present, negatively affected the network connectivity in SIBO individuals (Valiente-Banuet et al. 2020).

**Table 7.** Reported taxonomical changes (phylum and genus level) in the small intestinal regions between SIBO and healthy individuals.

			Phylum		Genus/family		
Location	Subjects (number)	SIBO diagnostic tool	Relative increase in SIBO	Relative decrease in SIBO	Relative increase in SIBO	Relative decrease in SIBO	References
***Duodenum***							
Lumen	SIBO (42) Non-IBO (98)	Aspirate culturing. Bacterial counts ≥10^3^ CFU/ml (on MacConkey agar)	Pseudomonadota	Bacillota Actinomycetota Fusobacteriota TM7	*Klebsiella* *Escherichia–Shigella* Unknown genus from Aeromonadaceae unknown genus from Moraxellaceae	–	Leite, et al. (2020a)
	SIBO (66) Non-IBO (60)	Aspirate culturing. Bacterial counts ≥10^5^ CFU/ml (aerobic, anaerobic, or both)	–	–	–	SIBO did not correlate with intestinal dysbiosis. Symptomatic patients: *Prophyromonas* *Prevotella* *Fusobacterium*	Saffouri et al. (2019)
Lumen Mucosa	SIBO (15) Non-IBO (21)	Aspirate culturing. Bacterial counts: ≥10^4^ CFU/ml Gram-negative aerobic or anaerobic bacteria OR ≥10^5^ CFU/ml Gram-positive aerobes, facultative aerobes, or other microbes from proximal gut and oropharynx	– –	– –	Coliform SIBO: *Granulicatella* spp. *Clostridium sensu stricto* Coliform SIBO:Clostridium spp.	– –	Shin et al. (2019)
Mucosa	SIBO (17) Non-IBO (14)	H_2_ and CH_4_ breath testing with lactulose	–	–	unknown genus from Absconditabacteriales	*Lactobacillus* *Prevotella_1* *Bifidobacterium* *Dialister* unknown genus from *Ruminococcaceae* *Clostridium sensu stricto 1*	Kuang et al. (2021)
***Ileum***							
Mucosa	SIBO (17) Non-IBO (14)	H_2_ and CH_4_ breath testing with lactulose	–	–	*Enterococcus* *Sutterella* *Holdemanella Butyricimonas Ruminococcus torques* group	*Lactobacillus* *Prevotella* unknown genus from *Chloroplast Clostridium sensu stricto 1* *Dialister* *Ruminococcus gnavus* group, unknown genus from Ruminococcaceae *Butyrivibrio* *Ruminococcus* unknown genus from Xanthobacteriaceae *Agathobacter* *Rhodococcus* *Klebsiella*	Kuang et al. (2021)

However, the efforts in characterizing the small intestinal ecology in SIBO patients may result in diverging results due to the difference in sampling locations across different study cohorts (Leite et al. 2020a, Li et al. 2021) (Table 7). Additionally, inconsistencies among SIBO studies might also be related to the type of SIBO, often not indicated. Shin et al. (2019) described two subtypes of SIBO, based on the type of bacteria overgrowing: upper aerodigestive tract SIBO and coliform SIBO, related to upper gut and oropharynx or colon-like bacteria, respectively (Table 7). When comparing these SIBO groups a significant difference in β-diversity is described. Additionally, when compared to non-SIBO individuals, significant taxonomical differences were described only between coliform SIBO type and non-SIBO individuals (Shin et al. 2019). These results indicate the need for a better microbial characterization of SIBO patients, including a more tailored discrimination between the two aforementioned SIBO groups, potentially determining different management strategies, as also suggested by other authors (Saffouri et al. 2019, Barlow et al. 2021).

Current methods to manage SIBO are mainly empirical and include antibiotics and a change in diet. The goal of a conventional therapeutic approach with antibiotics is to modulate, and at least partially inhibit the microbial community to eventually improve symptoms (Quigley et al. 2020). Rifaximin, a broad-spectrum nonabsorbable antibiotic, often prescribed for SIBO, shows *in vitro* efficacy against bacteria often associated with SIBO among which *Klebsiella* spp., *E. coli*, and *Enterobacter* spp. (Pistiki et al. 2014) making it possibly more effective in coliform SIBO. Although proven *in vitro* and *in vivo* efficacy, with SIBO eradication in about 70.8% of patients, (Gatta et al. 2017, Barkin et al. 2019), reoccurrence of SIBO after antibiotic treatment is common. In a study group treated with rifaximin, about 44% relapsed within 9 months (Lauritano et al. 2008). The high relapse number might be due to the underlying cause, which is not always possible to cure. Alternatively, dietary intervention with a reduced intake of poorly absorbable short-chain carbohydrates (fermentable oligosaccharides, disaccharides, monosaccharides, and polyols) as in low-FODMAP diets, used to treat SIBO patients, however, more evidences on its effectiveness and mechanisms are required (Srisukthaveerat et al. 2021, Biesiekierski and Tuck 2022, Wielgosz-Grochowska et al. 2022). Additionally, the use of probiotics in SIBO was shown effective in SIBO eradication and symptom relief according to a meta-analysis (Zhong et al. 2017), yet there is no consensus and little detail regarding the used probiotic strains and doses. In an attempt to approach SIBO treatments in a more holistic and ecosystem-oriented approach, fecal microbial transplants are also considered for modulating the gastrointestinal community. This has already been proved as effective strategy to treat recurrent *Clostridium difficile* infections (Fuentes et al. 2014). In SIBO, fecal microbial transplants resulted in an improvement of gastrointestinal symptoms (Xu et al. 2021), though more research is needed to confirm the result of this trial.

## Models of the small intestinal environment

**Figure 2.**
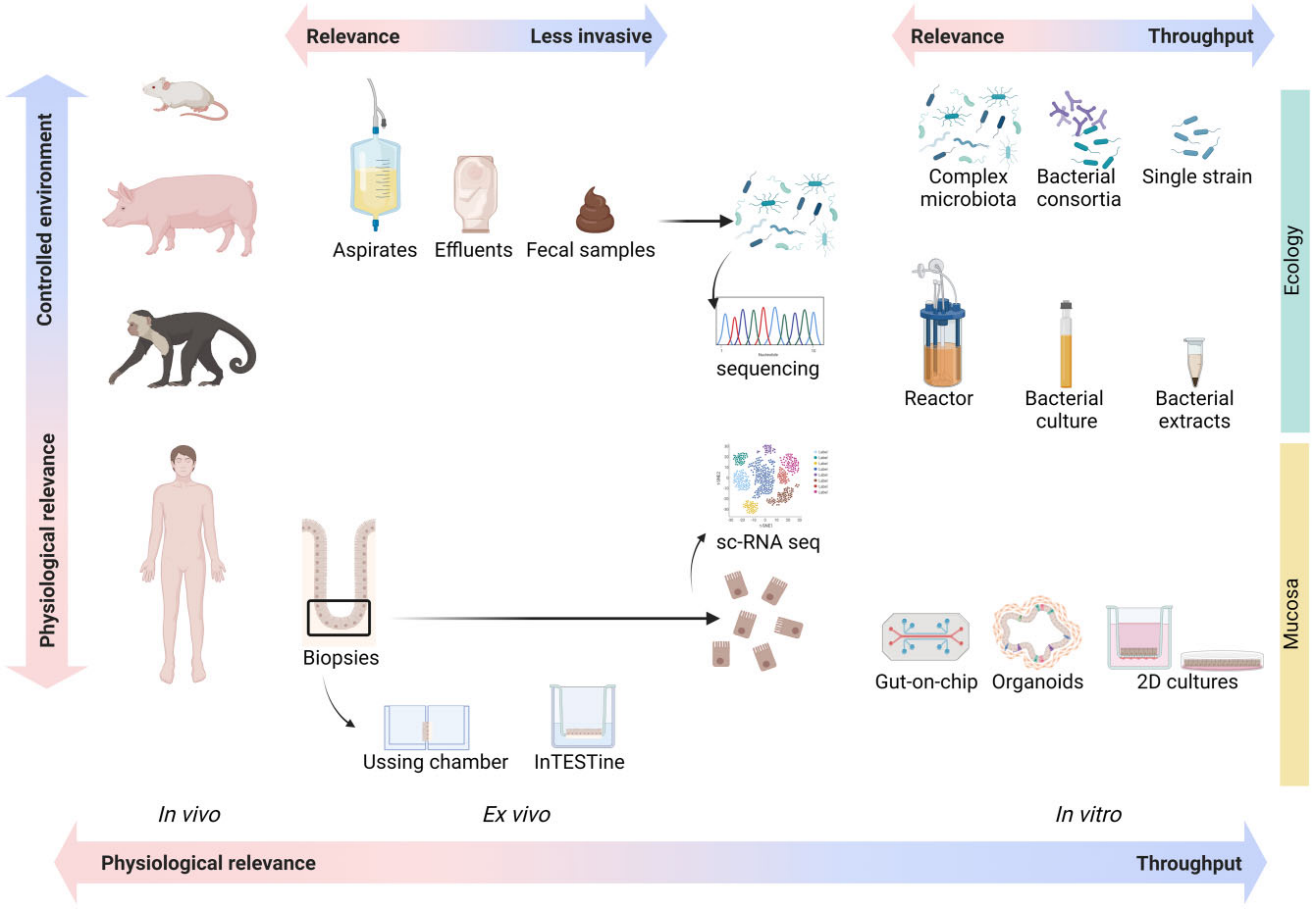
Overview of *in vivo, ex vivo*, and *in vitro* models used in the context of small intestinal studies. Major limitations and advantages in term of invasiveness and physiological relevance are indicated. Created with BioRender.com.

To untangle the complexity of the intestinal environment, *in vivo, ex vivo*, and *in vitro* approaches have been designed and employed to address the microbial ecology, intestinal mucosa, and the host–bacteria–nutrition interactions. In this section, we discuss the models used to mimic the adult healthy small intestinal environment and employed to study host–bacteria–nutrition interaction (Fig. 2).

### Human studies

In humans, medical practices like biopsies, luminal brush, catheter aspiration, and intelligent wireless capsules, allow to access and sample the small intestine *in vivo*. However, when sampling in *in vivo* conditions, the invasiveness of the medical practice, might prevent sampling from healthy individuals. Additionally, study subjects are often of higher age or with an underlying disease, which urges them to perform an endoscopy, surgery, or colectomy (removal of the colon) giving researchers the opportunity to sample. Consequently, some bias linked to the sampling method and study population, is introduced and makes the comparison of results from different cohorts challenging. Alternatively, samples from sudden-death victims and ileostomy patients offer valuable information to investigate *in vivo* features of the small intestinal environment with limited cross-contamination. Individuals with an ileostomy (i.e. no colon) allow repeated sampling of small intestinal content over time (Booijink et al. 2010, Zoetendal et al. 2012, Jonsson 2013, Van den Bogert et al. 2013, Van Trijp et al. 2020). However, in these cases, the risk of inaccurate representation of the small intestinal microbiota exists, due to the abnormal anatomy of the gastrointestinal tract (i.e. absence of colonic reflux) and suggested increased oxygen penetration (Hartman et al. 2009, Booijink et al. 2010, Zoetendal et al. 2012). Yet, a comparison of the small intestinal microbiota from healthy and ileostomy individuals showed ileostomy effluent clustered closely to jejunal samples, and ileum samples from healthy adults positioned between the ileostomy effluent and fecal samples (Zoetendal et al. 2012). Moreover, the penetration of oxygen is suggested to be limited as strict anaerobes, such as *Ruminococcus gnavus* and *Coprococcus eutactus*, are still detected in ileostomy effluents (Booijink et al. 2010, Zoetendal et al. 2012).

Sampling sudden death victims is also prone to sampling-bias (Hayashi et al. 2005). In particular, these studies often consist of elderly individuals, and aging has been shown to impact the small intestinal microbiota with reduced α-diversity and increased *Pseudomonadota* levels (*Enterobacteriaceae, Escherichia*, and *Klebsiella*) (Leite et al. 2021). In addition, *post mortem* microbial changes can occur as a result of, among others, hypoxia and nutrient depletion (Tuomisto et al. 2013). Yet, a study by Pechal et al. (2018) demonstrated that microbiomes from mouth and rectum can still represent the *ante mortem* health conditions within 24–48 h of death, but, to our knowledge, this has not been validated for small intestinal composition. As such, a timely sampling (within hours after death, as suggested by Hayashi et al. 2005), is crucial to limit the variability in the microbial composition.

An alternative to the aforementioned sampling conditions, is offered by directly accessing the small intestine, during gastric surgery or a cystectomy (Villmones et al. 2018, 2022). In patients undergoing a cystectomy, the distal ileum community was found to be similar to the oral community (Villmones et al. 2018). These findings are in line with what was described in sudden death victims (Hayashi et al. 2005), but contradicting other studies sampled through colonoscopy, which found increased presence of Bacteroidota, with higher similarity to the colon community (Wang et al. 2005, Vuik et al. 2019, Nagasue et al. 2022). Additionally, cross-contamination during sampling, can also explain for these differences. Indeed, while in Hayashi and Villmones reports they accessed the small intestine directly during surgery (Hayashi et al. 2005, Villmones et al. 2018, 2022), in other studies the authors made use of antegrade or retrograde endoscopy (Wang et al. 2005, Zoetendal et al. 2012, Dlugosz et al. 2014, Li et al. 2015, Sundin et al. 2017, Saffouri et al. 2019, Seekatz et al. 2019, Vuik et al. 2019, Fan et al. 2020, Kashiwagi et al. 2020, Vaga et al. 2020, Leite et al. 2020b, Barlow et al. 2021, Nagasue et al. 2022), with higher chance of cross-contamination by the densely populated mouth or colon environment. Furthermore, while preparation for an antegrade endoscopy usually requires only an overnight fasting period, for retrograde endoscopy (colonoscopy), bowel preparation by use of a laxative is often demanded to enable a clear view during colon inspection (Dlugosz et al. 2014, Sundin et al. 2017, Saffouri et al. 2019, Vuik et al. 2019, Fan et al. 2020, Kashiwagi et al. 2020, Vaga et al. 2020, Barlow et al. 2021). Bowel preparation is a well-documented source of bias for the description of intestinal microbial composition *in vivo* in humans (Shobar et al. 2016, Nagata et al. 2019). However, the extent to which it affects the small intestinal ecology has not yet been reported.

As described, all above mentioned techniques are not suitable for healthy controls, due to the invasiveness of the procedure and the associated ethical restrictions. To overcome this limitation, intelligent wireless capsules have been developed to monitor parameters in the intestinal environment and, in some cases, sample the lumen content, in healthy individuals without the need for invasive procedures (Tang et al. 2020). Yet, the high costs, the potential cross-contamination with sampling sites up- or downstream and the sample preservation until fecal excretion, still limit their applicability. To preserve the sample during the multiple hours the pills remain at body temperature, Rios-Morales et al. (2021) developed a quenching agent to stabilize microbial composition, fibers fermentation, and SCFAs production for 48 h, enabling studying the small intestinal microbial composition, yet limiting downstream culturomics approach.

### *In vivo* models

#### Animal studies

To study physiological processes, animal models are often considered a suitable alternative to human *in vivo* studies, offering the possibility to analyze multiple endpoints and the impact of genetic modifications. Additionally, germ-free animals provide the opportunity to study specific microbial populations in host–microbiota applications. Advantages and limitations of monogastric animal models, such as nonhuman primates, pigs, and rodents for the study of small intestinal ecology and host–bacteria interaction, are discussed here.

#### Nonhuman primates

Nonhuman primates are our closest relatives and, therefore, a highly relevant research animal model. However, the expensive and difficult housing and husbandry of nonhuman primates, along with strict ethical regulations, limit a more widespread employment as *in vivo* model (Walker and Eggel 2020). In the context of host–microbe interaction research, different nonhuman primate models exist: (i) wild nonhuman primates, with distinct microbial ecology, different to humans; (ii) captivated nonhuman primates (Firrman et al. 2019, Yuan et al. 2020, Yan et al. 2022), showing microbiota resembling more to human microbiome than wild nonhuman primates (Clayton et al. 2016). To assess the impact of multiple diets on the human microbiome, captivated nonhuman primates fed a human-like diet during the study, are often employed (Amato et al. 2015, Nagpal et al. 2018, Newman et al. 2021). However, despite the genetic proximity with humans, not all nonhuman primate species are suitable for extrapolation to human gut microbial research. For example, compared to humans there are marked differences in the intestinal microbial community for capuchin monkeys (*Cebus apella*), (Firrman et al. 2019), characterized by lower relative abundance of *Streptococcus* in small intestine. In baboons as well, *Spirochaetes*, not commonly found in the human gut (Angelakis et al. 2019), Bacillota, Bacteroidota, and Pseudomonadota (in consecutive order) are described to be the main species in the small intestine (duodenum, jejunum, and ileum) (Yuan et al. 2020). Conversely, in rhesus macaques, there is a lower presence of *Spirochaetes* in the mucosa and a predominance of Bacteroidota, Bacillota, and Pseudomonadota in the gut (Yasuda et al. 2015). Nonetheless, the relevance of nonhuman primates for direct testing host–microbe–nutrition interactions was questioned, as similar diets are described to differently affect the human and nonhuman primate gut microbiota based on the rectal microbiota (Amato et al. 2015).

#### Pigs

Pigs are an alternative *in vivo* model, with high relevance to human gut anatomy, physiology, nutritional requirements, and immune system (Meurens et al. 2012). One of the advantages of the pig model is the possibility for cannulation (often performed in the terminal ileum), enabling multiple sampling points over time within the gastrointestinal tract, without need for euthanasia (Metzler-Zebeli et al. 2010, Shen et al. 2020).

To study the host–microbe–nutrition interaction, multiple approaches in the pig exist, among which: (i) conventional pigs (i.e. with conventional pig feed and microbiome), as beside a relevant model for human health, there is a concern in pigs health by their economic value (Zhao et al. 2015, Crespo-Piazuelo et al. 2018, Zhang et al. 2018); (ii) germ-free pigs which offer the possibility to study specific bacterial communities including the complex human microbiota by fecal microbial transfer (human-microbiota associated pigs), though no comparison to the small intestinal microbiota has yet been published to our knowledge (Aluthge et al. 2017, 2020, Fischer et al. 2017, Zhou et al. 2021). To study microbe–nutrition interaction in the human context, pigs can be fed with a human-like diet, comprising foods commonly consumed by humans (Hoogeveen et al. 2020, Shen et al. 2020, Xu et al. 2020). In conventional pigs, the small intestine (duodenum, jejunum, and ileum) is dominated by Bacillota and Pseudomonadota (Zhao et al. 2015, Crespo-Piazuelo et al. 2018), while the presence of Pseudomonadota increases throughout the small intestine reaching maximum presence in the ileum, similar to what was observed in humans. Yet, at lower taxonomic levels discrepancies with the human microbial community remain present (Crespo-Piazuelo et al. 2018, Nagasue et al. 2022). One element that should be taken into account regarding intestinal stressors that impact resident small intestinal microbiota, is the difference in bile salt profiles between pigs and humans. Indeed, the two major constituents of human bile salts, cholic, and deoxycholic acids, are present at lower proportion in pigs. Conversely, hyocholic and ursodeoxycholic acids, minor bile salts in humans, represent a higher fraction in pigs (Spinelli et al. 2016).

#### Rodents

The use of rodents (mostly mice and rats) in gut microbiome studies is well-established thanks to their reduced housing and husbandry costs (in comparison to nonhuman primates and pigs), high reproductive rates, short life cycle, and possibility for genetic manipulations. Moreover, the availability of well-established transgenic mouse strains for several human diseases, has driven the increased interest of this model for research, during the last decade (Flemer et al. 2017). In contrast, the bigger size of rats permits increased sampling (tissue and feces) and they are reported to sustain human-like fecal microbiota profiles better than mice (Flemer et al. 2017, Lleal et al. 2019).

In host–bacteria–nutrition interaction studies, multiple rodent models can be considered, by adjusting genetics (e.g. gene knockouts), gut microbiota composition (e.g. germ-free, mono-colonized gnotobiotic, humanized gnotobiotic, specific pathogen free, or conventional) or environment (e.g. diet, antibiotics) (Martinez-Guryn et al. 2018, Chen et al. 2020, Todorov et al. 2020, Tuganbaev et al. 2020, Escoto et al. 2021). Choosing the adequate model and housing condition for the scientific question to address, is crucial. Indeed, animals born and raised in total sterile conditions, show altered physiology, including extensive deficit in the development of mucosal immunity (Round and Mazmanian 2009), reduced number of Paneth cells in (jejunal) mucosa associated with decreased concentration of antimicrobial peptide secretion (Schoenborn et al. 2019), and a major susceptibility to chemically induced epithelial damages (Hayes et al. 2018). Conversely, the antibiotic-induced germ-free condition, may result in only a partial depletion of bacterial species, induces antibiotic-resistance, and impacts epithelial cell metabolisms (reviewed by Kennedy et al. 2018).

Instead of regular fecal microbial transplants for humanization of germ-free mice, Li et al. (2020a) explored the introduction of whole intestinal microbial transplants, comprising not only fecal bacteria but also microbiota from jejunum, ileum, cecum, and colon (derived from pigs’ intestine) and observed an increased colonization of small intestinal related bacteria in the small intestine. Based on this study, whole intestinal microbial transplants would be a more representative alternative for rodent humanization in view of small intestinal research (Li et al. 2020a).

For small intestinal microbial research, the use of rodents has been questioned by their habit of coprophagy described to shift the small intestinal microbiota closer to the colon composition, with increased presence of *Clostridiales* and *Bacteroidales*, while noncoprophagic mice, prevented by wearing tail-cups, were dominated by lactobacilli, a taxon also regularly found in the humans small intestine (Hayashi et al. 2005, Li et al. 2015, Seekatz et al. 2019, Bogatyrev et al. 2020). This questions the relevance of mouse models in small intestinal microbial research and, to our knowledge, has not yet been addressed in current studies. However, tail-cups can induce stress to the animals (observed by a decreased weight) and it is described to influence the gut microbial community (Gao et al. 2018), resulting in the introduction of an additional bias for microbiome studies. In the study of Bogatyrev et al. (2020), the microbial community and bile acid profiles between mock-tail-cup (i.e. a tail cup which does not prevent coprophagy) and coprophagic animals was similar, suggesting possible stress induced by the tail-cup does not influence the upper gastrointestinal tract community significantly. No comparable studies investigating the impact of coprophagy on the small intestinal microbiota of rats are published, though the fecal community of rats wearing a tail-cup shown a decreased presence of lactobacilli and increased enterococci and coliforms (Fitzgerald et al. 1964). Nonetheless, a distinction in the microbial community of rodents between the small intestine and lower gastrointestinal tract is still found (Gu et al. 2013, Li et al. 2017). Another possible confounder in the use of rodents as *in vivo* model is that mice and rats are largely herbivorous, with an intestinal anatomy adapted to this feature (i.e. large cecum). This may determine different ecological selection processes, resulting in a proportionally higher abundance of fiber-degrading microbiota in the rodent gut as opposed to the human gut.

The extrapolation of host–microbiota findings from animals to humans should be performed with care. The cospeciation between mammals and gut microbiota, whether or not as a result of coevolution, host biogeography, or allopatry (Groussin et al. 2020), results in symbiotic interactions, likely resulting in different responses or decreased microbial colonization if studied in other animals (Chung et al. 2012, Amato et al. 2015, Lundberg et al. 2020). In this view, when studying host–bacteria–nutrition interactions, the model should be chosen based on the scientific question and ultimately, if possible, the best model remains humans, despite increased ethical constraints and sampling difficulties.

### *Ex vivo* models

One of the ways to study host–microbe–nutrient interaction in the small intestine is to make use of *ex vivo* intestinal explant tissues, derived from either human or animal small intestinal biopsies and exhibiting a high resemblance to *in vivo* tissue complexity and morphology (Rozehnal et al. 2012, Roeselers et al. 2013). *Ex vivo* intestinal tissue segments from the different regions of the small and large intestine allow the investigation of regional absorption and immune responses (Rozehnal et al. 2012).

Different *ex vivo* approaches have been developed during the years to study host–microbiota interaction in small intestine, the most prominent being the Ussing chamber and the InTESTine^TM^ System. The Ussing chamber has become a useful *ex vivo* tool widely used to assess the transport of several materials and nutrients (i.e. glucose, amino acids, and minerals) across different segments of the intestinal tract. This model has also been applied to study host–microbe interactions largely in the context of altered or damaged permeability, such as in the case of exposure to bacterial toxins but also with live intact bacteria. However, the application of Ussing chamber to study host–microbiota interaction in healthy condition is scarce and mostly restricted to colonic epithelium. Although being an excellent tool to study intestinal permeability, a major limitation for the application of Ussing chamber is that the epithelial layer alone is not capable of fully recapitulating the *in vivo* complexity. Additionally, the difficult preservation of the tissue viability through the experimental period (restricted to a maximum of 90 min, as tested with human ileum tissue in Söderholm et al. 1998) limits its applications to short-time measurements. Lastly, another major drawback of the Ussing chamber is its low-throughput, since it does not allow simultaneous preparation and analyses of a large set of segments of epithelial tissues, limiting its applicability in the context of compound screening. Nonetheless, Ussing chamber have been employed to study intestinal transport and barrier function after lipopolysaccharide exposure (Albin et al. 2007), nutrient supplementations (Woyengo et al. 2012) in animal small intestine and the impact of bacterial invasion (Isenmann et al. 2000, Jafari et al. 2016), and probiotics (Chen et al. 2010, Shi et al. 2014), in colon. Alternatively, The Netherlands Organization for Applied Scientific Research (TNO) has recently developed an *ex vivo* tissue model called the InTESTine™. Like in the Ussing chamber, fresh intestinal tissue (duodenum, jejunum, ileum, and colon) of human or porcine origin are mounted in the two-compartment system creating an apical and basolateral side (Westerhout et al. 2014, Stevens et al. 2019). Compared to Ussing chamber, the InTESTine™ system provides a higher throughput and easy horizontal setup in standard 6- or 24-well plates *via* which up to 96 *ex vivo* intestinal tissue can be used per day to test for intestinal absorption.

### *In vitro* models

The host–microbiome research field has greatly benefit from the development of *in vitro* models. By simulating the physiological conditions in a controlled environment and limited confounding factors, *in vitro* models have been widely developed and used to address different scientific questions, among which studying the complexity of the intestinal environment. Moreover the versatility, reproducibility, cost, and time-efficiency of *in vitro* models makes them well-suited for mechanistic studies and adaptable (most of the time) for high-throughput approaches. However, the fitness of use of *in vitro* models depends on the scientific question. In the next section we describe *in vivo* models, their limitations, and advantages, when used to study small intestinal ecology and mucosa.

#### In vitro models for small intestinal microbiota

*In vitro* culturing of the small intestinal microbiota was firstly addressed by using static models, such as the small intestinal model developed by Schantz et al. (2010). Through a batch set-up inoculated with ileostomy effluent in airtight anoxic tubes, shaken and incubated at 37°C for 24 h, the authors studied the impact of ileal microbial digestion on the green tea catechins and described interindividual differences between donors in both aerobic (*E. coli, Proteus* sp., and *Enterococcus* sp.) and anaerobic bacterial species (*Bacteroides* sp., *Bifidobacterium* sp., and *Lactobacillus* sp.) and fungi (*Geotrichum* sp.). Batch models are suitable *in vitro* models to screen various treatments in small reactors during a short time-span and with limited costs, but they remain an oversimplification of the *in vivo* situation, often without pH regulation or transit time simulation. In addition, such an *in vitro* method is mainly limited by the invasiveness of ileostomy, which cannot be performed on healthy volunteers.

To overcome these limitations, dynamic (semi-)continuous long-term culture approaches were developed, reproducing the main physico-chemical parameters of the human gut (i.e. pH, temperature, retention time, nutrient availability, and anoxic conditions). Existing dynamic (semi-)continuous *in vitro* models have been mainly developed and validated to study the colon microbial community {TIM2® [TNO gastro-Intestinal tract Model (Stolaki et al. 2019)], ARCOL [Artificial Colon (Deschamps et al. 2020)], ECSIM [Environmental Control System for Intestinal Microbiota (Brugère et al. 2011)], and SHIME® [Simulator of the Human Intestinal Microbial Ecosystem (van de Wiele et al. 2015)]}, though by the increasing evidence of the importance of the small intestinal microbiota to human health (Aidy et al. 2015), some of these were adapted to study the small intestinal ecology.

The study of the ileal ecosystem with dynamic *in vitro* models is, so far, primarily achieved through three bioreactor models: (i) The Smallest Intestine (TSI) (Cieplak et al. 2018); (ii) the SHIME® (Roussel et al. 2020), and (iii) the TIM2® (Stolaki et al. 2019). These three dynamic models implement continuous pH regulation, maintain anoxic conditions, and simulate the transit time of each studied part. They mainly differ in what part of the intestinal tract is studied, and how the ileal microbiota is introduced into the model (summarized in Table 8). In the context of SHIME® experiments, fecal microbiota from human origin were first adapted to proximal colon conditions. Then, the retrograde microbial colonization through the ileocecal sphincter, was simulated by a diluted feedback inoculation from proximal colon to the terminal ileum compartment (Laird et al. 2013, Roussel et al. 2020). This approach resulted in lower diversity and metabolic activity in the ileal compartment (assessed by SCFA quantification) compared to the ascending colon, yet some key taxa characteristic of the small intestinal microbiota, such as *Streptococcus*, remained absent or were present in low numbers (Roussel et al. 2020).

**Table 8.** Global table of dynamic *in vitro* gut models including small intestine part and using microbiota. This table summarizes the parameters used in each model and the context of the study. Duo = Duodenum; Jej = jejunum; Il = Ileum. GI = gastrointestinal; TIM = TNO gastro-intestinal tract model; (M-)SHIME = (Mucosal) Simulator of the Human Intestinal Microbial Ecosystem; and TSI = The Smallest Intestine.

Model name	Origin of microbiota	Simulated GI compartments	Transit time	pH	Application/context	Anaerobic condition	References
TIM-2	Ileostomy effluentor fecal inocula	Ileum	3.5 h	7.2	Model development	Nitrogen flush	Stolaki et al. (2019)
M-SHIME	Fecal sample in colon used to inoculate ileum at days 3 and 8	Stomach to ascending colon	Duo = no value Jej = no value Il = 3 h	Duo = no value Jej = no value Il = 6	ETEC colonization and virulence	Nitrogen flush	Roussel et al. (2020)
SHIME	Fecal sample in colon used to inoculate ileum part of static SHIME	Stomach to colon	Duo = 6h Jej = no value Il = 3, 4 or 5 h	Duo = 6.5 Jej = no value Il = 6.8	Arsenic bioavailability	Nitrogen flush	Laird et al. (2013)
SHIME	*Enterobacteriaceae* *Enterococci* Lactic acid bacteria *Clostridium spp*.	Stomach to colon	Duo / Jej = 4 h Il = 4 h	Not controlled	Effect of probiotic on microbiota	Nitrogen flush	Alander et al. (1999)
SHIME/static digestion	Fecal sample in ascending colon used to inoculate ileum	Stomach to ascending colon	Duo = 2 h Jej = no value Il = 4 h	Duo = 6.5 Jej = no value Il = no value	*Bacillus cereus* growth and sporulation	Microaerobic conditions in sealed glass bottles	Ceuppens et al. (2012)
TSI	Seven bacteria in ileum *E. coli/S. salivarius/S. luteinensis/E. faecalis/B. fragilis/V. parvula/F. plautii*	Duodenum Jejunum Ileum	Duo = 2h Jej = 4h Il = 2 h	Duo = 6.5 to 6.8 Jej = 6.8 to 7.2 Il = 7.2	Model development	Nitrogen flushor Anaerobic sachets	Cieplak et al. (2018)
TSI	Seven bacteria in ileum*E. coli/S. salivarius/S. luteinensis/E. faecalis/B. fragilis/V. parvula/F. plautii*	Duodenum Jejunum Ileum	Duo = 2h Jej = 4 h Il = 2 h	Duo = 2 h Jej = 4 h Il = 2 h	Effect of a bacteriophage cocktail on *Listeria monocytogenes*	Nitrogen flushor Anaerobic sachets	Jakobsen et al. (2022)

Efforts to compare ileostomy effluents and fecal samples as inoculum in the ileum compartment, on a TIM device, resulted in the description of similar communities developed independently from the used sample but distinct from the original inoculum, and mainly constituted of Bacteroidota, Pseudomonadota, Bacilli, *Clostridium*, and Actinomycetota (Stolaki et al. 2019). Additionally, in this report, the authors described the presence of *Streptococcus*, in low amounts. In contrast, to have a straightforward and easily reproducible ileal microbiota, TSI does not require ileal or fecal samples to inoculate, and is rather inoculated with a consortium of seven bacteria, known to be present in the human small intestine: *E. coli, S. salivarius, S. luteinensis, Enterococcus faecalis, Bacteroides fragilis, Veillonella parvula*, and *Flavonifractor plautii* (Cieplak et al. 2018, Jakobsen et al. 2022). By simplifying the complexity of the ileal microbiota, this model avoids the study of interindividual variability, therefore, limiting extrapolation to the physiological condition. In addition, this model requires several days to prepare the consortium with different incubation times of the bacteria and does not reproduce the complex interactions occurring within the small intestinal microbiota ecosystem. Nevertheless, one of the main advantages of the TSI model is to allow a better throughput by using five low volume ileal vessels in parallel.

Despite the existence of these *in vitro* models to mimic the small intestine, the development and the validation of physiologic-like bioreactors able to simulate the small intestinal microbial community remains challenging. This is mostly due to ethical constrains, restricting the accessibility for an appropriate inoculum from healthy donors to validate the *in vitro* model against *in vivo* condition, along with technical issues to manage oxygen levels, impacting obligate aerobe and facultative anaerobe in the ileum community. Additionally, the small intestine is a low microbiota biomass environment with low diversity and quantity compared to the high stool microbial biomass. This increasing load of bacteria along the small intestine, as well as changes in microbial profiles are challenging to reproduce in an *in vitro* model, as well as the human interindividual differences. In order to address interindividual differences, some studies on colonic microbiota indicate parameters such as transit time and nutrient load are important drivers for microbial community development (Vandeputte et al. 2017, Minnebo et al. 2021, Procházková et al. 2022).

To date, very few information exists on *in vitro* microbial models simulating the ileal ecology and, to our knowledge, none for the duodenum or jejunum. Increased validation and research are essential to unlock the potential of *in vitro* systems in this still understudied area.

#### In vitro models for intestinal mucosa

In order to study the regulation of intestinal epithelial homeostasis and the mechanisms of this fine regulating host-environmental cross-talk, different epithelial models have been developed. Depending on the research question (study nutrient absorption, permeability, and host–microbe interactions) different levels of complexity to these models are needed.

The challenges of developing an *in vitro* model suitable to study host–microbiota–diet interactions resides in the combination of the key components constituting such models: (i) the epithelial cell layer, ideally including different cell type to recapitulate diverse epithelial functions; (ii) the microbial component, and (iii) eventual dietary compounds and additional compartments, mimicking other epithelial functions (e.g. immune, enteroendocrine, and secretory functions). Current *in vitro* small intestinal cellular models, aimed at studying nutrient absorption and host–microbiota interaction, range from simple 2D systems, such as cocultured intestinal cells on microporous Transwell^®^ supports (Hidalgo et al. 1989, Hilgers et al. 1990), to more complex 3D models with coculturing techniques that enable more than one type of intestinal cell (i.e. mucosal epithelia and a submucosal cell type) to be incubated simultaneously with select bacterial populations (Bernardo et al. 2012). Indeed, it is well known that the 3D physical environment plays a major role in the morphology, biochemistry, and metabolism of mammalian cells (Anselme and Bigerelle 2006, Bettinger et al. 2009). Yet, mimicking the complex 3D crypt–villus architecture remains challenging.

To simulate the cellular component *in vitro*, well-established immortalized cell lines, primary cell cultures, or induced pluripotent stem cells (iPSCs) can be used. In the next section, we describe several *in vitro* cell models developed for studying nutrients absorption and host–microbiota interaction in the small intestine.

#### Immortalized cell lines

Immortalized cells are derived from a population of cells that have evaded normal cellular senescence due to a mutation and are therefore able to proliferate indefinitely.

To mimic enterocytes, human Caco-2, HT-29 (both derived from colon adenocarcinoma) and HuTu-80 (derived from duodenum adenocarcinoma) cell lines are generally used *in vitro* models to predict absorption, to study permeability and diffusion of compounds through the epithelium, and describe mechanisms of host–microbe interactions (Hidalgo et al. 1989, Smetanová et al. 2011, Brosnahan and Brown 2012, Kavanaugh et al. 2013, Takenaka et al. 2016). In particular, Caco-2 cells are a well-established model for enterocytes and are, therefore, incorporated in many *in vitro* models. After seeding, Caco-2 cells spontaneously differentiate to form confluent monolayer of polarized cells, structurally and functionally resembling the small intestinal epithelium and expressing several morphological and functional properties characteristic of small bowel enterocytes. In particular, Caco-2 cells have been found to express a large number of enzymes and transporter proteins present in normal human small intestinal epithelium. For example, exposing Caco-2 cells to fluidic shear or periodic contraction, induces the expression of metabolic enzymes, mucus proteins, as well as formation of villus-like structures (Kim and Ingber 2013, Lindner et al. 2021). However, recent studies suggest that variations exist between gene expression profiles of transformed epithelial cell lines, like Caco-2, and normal human intestinal epithelium (Bourgine et al. 2012), limiting their physiological relevance. Indeed, although widely used, immortalized cells are generally derived from cancer cells and, as such, metabolically differ from intestinal cells in healthy condition.

Several mucin-producing immortalized cell lines have been established and used to mimic goblet cells, in the context of the small intestinal research. For example, the colonic cancer tissue isolated LS174T cell line shows high *MUC2* mRNA expression, in line with the secretory profile of small intestinal goblet cells, along with low *MUC5AC* expression and are, therefore, often used as a model for goblet cells (Lesuffleur et al. 1990, van Klinken et al. 1996, Martínez-Maqueda et al. 2013).

Besides LS174T cells, the HT29-MTX cell line is commonly used in *in vitro* models to simulate goblet cells phenotype. This cell type originates from the gradual exposure of the human colorectal adenocarcinoma HT-29 cell line to increasing concentrations of methotrexate (MTX), resulting in their transformation into mucus-secreting differentiated cells (Lesuffleur et al. 1990). The major secreted mucin in this cell line is MUC5AC, along with the in lesser amounts-secreted MUC2 (Martínez-Maqueda et al. 2013, Elzinga et al. 2021).

To address the enteroendocrine function of the small intestinal epithelium, mammalian EEC lines, such as human NCI-h716 cell line, are widely used to study gut hormone response to environmental stimuli, including food and microbiota. NCI-h716 cell line exhibits lymphoblast morphology and enteroendocrine differentiation, including the expression of secretory granules and chromogranin A (De Bruine et al. 1993). This cell line produces GLP-1, GLP-2 (Kuhre et al. 2016), making it suitable to study the secretion of these hormones. However, it does not produce CCK nor peptide-YY (PYY) (Kuhre et al. 2016), limiting its resemblance with normal L-cells. Alternatively, another widely used human cell line expressing PYY and precursor of glucagon/GLP-1, is the HuTu-80, isolated from healthy human duodenal cells (Nevé et al. 2010). However, this cell line also expresses the glucose-dependent insulinotropic polypeptide (GIP), described as exclusively produced by K cells, *in vivo* (Rozengurt et al. 2006).

#### Immortalized cell lines in host–microbiota interaction studies

To better simulate the cellular complexity of the small intestinal epithelium and study the host–bacteria interaction, coculture of different immortalized cell lines, with bacterial samples, is frequently used.

For example, bacterial–epithelial interactions have been studied using a Caco-2 cell monolayer system seeded on a porous membrane (Cruz et al. 1994) or through triple coculture of epithelial (Caco-2), goblet (HT29-MTX), and immune-like cells (THP-1) in physical contact and in combination with a synthetic microbial consortium of eight bacterial strains resembling the small intestine microbiome (Calatayud et al. 2019). However, one major drawback of this approach is that these experiments can be carried out only over a relatively short period of time before bacterial overgrowth leads to cell injury and death.

An alternative approach for studying host–microbiota interaction using cell lines, has been proposed by Marzorati and colleagues in 2014 with the development of the human−microbiota interaction module (HMI^TM^ module). By combining a SHIME reactor with Caco-2 cells, the HMI^TM^ module permits to evaluate the effect of both aerobic and anaerobic microorganisms on enterocyte-like cells under an oxygen gradient. Because of the absence of cell-derived mucus in this model, a nanoporous membrane with an artificial mucus layer was added to separate the microbial community from the compartment hosting the cells, and, the cocultures were maintained for 48 h (Marzorati et al. 2014). Although applied for studying colonic bacteria, this set-up offers the possibility of coupling cell models with a continuous simulator of intestinal ecology and simulate both bacterial adhesion and indirect host–bacteria interaction.

Despite the available tools and the rapidly expanding research field, *in vitro* studies on host–microbe interactions in the small intestine, combining immortalized cell lines with bacterial samples, still remains limited, compared to colonic models, possibly due to the slower gain of knowledge on the ecology of this body site, for long time.

#### Primary and induced pluripotent cells

For improved physiological relevance, compared to immortalized cell lines, primary cells and induced pluripotent stem cells (iPS cells or iPSCs) are used, offering also the possibility for a patient-specific approach.

Both primary cells, isolated from either human or animal mucosal small intestinal biopsy tissue, and iPSCs, can be expanded to organoids or incorporated in 2D and 3D set-ups, such as Transwell® or gut-on-a-chip models, in which they can be combined with either immune cells or a microbial strain or community (Kasendra et al. 2018, Yin et al. 2021).

Organoids are self-organizing 3D structures, composed of both multipotent tissue-specific stem cells and differentiated cell lineages, led to unprecedented opportunities to generate highly versatile *in vitro* models that closely mimic *in vivo* conditions.

To date, this technique has allowed to successfully generate organoids from all human gut segments (Sato et al. 2011, Meran et al. 2020), able to retain both segment-specific (Middendorp et al. 2014, Kraiczy et al. 2019, van der Hee et al. 2020) and donor-specific properties (Vlachogiannis et al. 2018, Elmentaite et al. 2020), making it a valuable tool for personalized medicine. Furthermore, the ability of expanding and freezing live organoids paved the way for the establishment of biobanks of live human intestinal organoids (reviewed in Perrone and Zilbauer 2021).

In the context of small intestinal research, biopsies of adults, children, or fetal small intestinal tissues have been successfully used to generate organoids (also called enteroids), for epigenetic, transcriptomic, or single-cell profiling (Howell et al. 2018, Elmentaite et al. 2020, Stewart et al. 2020). Additionally, human intestinal stem cell-derived enteroids, cocultured with human immune cells, were employed to study epithelial-immune cell interactions (Noel et al. 2017, Staab et al. 2020). Small intestinal organoids have been successfully employed to describe the positive impact on the intestinal epithelial homeostasis of conditioned media from mucin-degrading *Akkermansia muciniphila* (Lukovac et al. 2014, Kim et al. 2021), intestinal bacteria, such as *Faecalibacterium prausnitzii* (Lukovac et al. 2014), and probiotic, e.g. *Lactobacillus rhamnosus LGG* (Aoki-Yoshida et al. 2016). Moreover, mouse and human ileal enteroids were used to investigate the effect of conditioned media from *Bifidobacterium dentium* on serotonin production (Engevik et al. 2021).

One of the major challenges of working with organoids, in particular for host–microbiota interaction studies, is to access lumen in the classical apical-in configuration. Besides single bacterial strains, attempt of injecting complex microbial communities, such as human fecal samples, were also successful (Williamson et al. 2018) but, to our knowledge, only restrained to the colonic environment. While this classical configuration allows to have a lumen environment sufficiently hypoxic to support the survival of anaerobic bacteria, this requires technically challenging microinjections to expose the apical side to different stimuli (i.e. bacteria, chemicals). In this regard, alternative methodologies have been proposed to expose the apical surface of the epithelium and facilitate the accessibility. For example, to study the host–microbiota interaction in the small intestinal environment, 2D models have been implemented from sheared organoids or single cell suspensions (Puschhof et al. 2021). Alternatively, reversed cell polarity was achieved with swine jejunum-derived organoids by culturing them in a suspension culture system (Li et al. 2020b, Kakni et al. 2022). Lastly, an innovative engineered tube-shaped perfusable intestinal epithelia was developed from mouse adult intestinal crypts, suitable for long-term culture and for modelling host–microbes interaction (Nikolaev et al. 2020), paving the way for novel enteroids models for host–microbiota interaction.

## Future perspectives

Given the increasing interest on diet–host–microbiota interactions in the small intestine, along with the great expansion of omics approaches, it is reasonable to believe that our knowledge on this research field can only grow wider in the next years and eventually lead to novel therapeutic strategies to support human nutrition in both healthy and disease conditions. However, the accessibility and sampling of small intestinal luminal content and mucosa without bias, still remains a major challenge in the field. In this context, robotic sampling capsules pose interesting perspectives for collecting the human small intestinal microbiota *in vivo* (Cui et al. 2008, van der Schaar et al. 2013, Koziolek et al. 2015, Jin et al. 2019, Rezaei Nejad et al. 2019).

A further limitation to fully characterize the small intestinal ecology from a functional perspective is the lack of metabolomic data, matching microbiome results in large cohort studies. To fill this gap, metabolite prediction methods have been developed to predict individual metabolites solely based on microbiome metagenomic or amplicon sequencing data (Langille et al. 2013, Xie et al. 2021). *In silico* simulations, coupled with *in vitro* and *in vivo* data can contribute to mechanistically describe physiological and metabolic process, such as prediction of the glycemic index of biscuits using chicken duodenum (Priyadarshini et al. 2021), characterization of the precipitation kinetics of drugs, and the prediction of their concentration in small intestine after human oral administration (Hens et al. 2014, Kambayashi et al. 2016). Additionally, metabolic models can be used to predict growth patterns of gut microbiota when exposed to different nutrients (Singh et al. 2022). Genome-scale metabolic reconstructions coupled with metabolomic data and dietary composition, allow to study interspecies interactions (Magnúsdóttir et al. 2015, Singh et al. 2022). Furthermore, protein–protein interaction bioinformatic workflows, based on intestinal microbiota composition, are available, combining metataxonomic analysis with domain-mediated protein–protein interaction approaches (Orsini et al. 2020).

Recent technological advances in cell culture technique and expanded knowledge in stem cell biology and bioengineering have driven a number of *in vitro* models integrating mucosal components and microbiota, to mimic the small intestinal environment and facilitate the characterization of mechanisms involved in this cross-talk. In this context, microfluidics-based techniques, such as organ-on-chip, and bioprinting technology are revolutionizing the field of *in vitro* organ modelling. Additionally, gut-multiorgan models, although still technically challenging, hold great potential for investigating the dialogue between the small intestine environment with distant organs, such as liver and brain.

To address absorption, distribution, metabolism, and elimination of drugs, an innovative gut–multiorgan model has been developed, incorporating (i) a human small intestinal compartment; (ii) a skin biopsy, for oral and dermal substance absorption; (iii) a liver component, for primary metabolism, and (iv) a kidney proximal tubule compartment, for metabolic excretion (Maschmeyer et al. 2015). As such, this model offers the great potential for effectively studying nutrient absorption, metabolism, and excretion along with the destiny of several other metabolites present in the small intestinal lumen, including those derived from intestinal microbes.

Besides its role in nutrient absorption, the small intestine harbors EECs and enteric nervous system that form an interface between the microbiota and the central nervous system. In this context, two innovative European-funded projects, IMBIBE (ID 723951 ‘Innovative technology solutions to explore effects of the microbiome on intestine and brain pathophysiology’) and MINERVA (ID 724734, ‘MIcrobiota–Gut–BraiN EngineeRed platform to eVAluate intestinal microflora impact on brain functionality’) have been designed to study the complex mechanisms of gut–brain axis on multiorgan-on-chip models. The IMBIBE project aims at exploiting conductive polymer 3D tubular scaffolds for continuous monitoring of cell activity and integrity of an integrated gastrointestinal and blood–brain barrier/neurovascular units (Pitsalidis et al. 2018, Moysidou and Owens 2021). Conversely, the MINERVA project makes use of five organ-on-chip devices to elucidate the effect of microbiota secretome on brain function, in a context of Alzheimer’s disease. In particular, the MINERVA platform is based on miniaturized organ-on-chip devices connected sequentially and designed to represent (i) the gut microbiota, (ii) the gut epithelial barrier, (iii) the immune system, (iv) the blood–brain barrier, and (v) the brain.

One of the great advantages of these organ-on-chip models is the possibility to adapt the model to different scientific questions and making them versatile for several applications, e.g. including the characterization of microbiota–small intestine–multiorgan axis.

Yet, besides these promising technological *in vivo, in vitro*, and *in silico* advances, collecting exhaustive information about compounds in the diet and the exact composition of macro- and micronutrients, might be challenging. To fill this gap, Blasco et al. (2021) developed AGREDA, an extended reconstruction of diet metabolism in the human gut microbiota, predicting diet-specific output metabolites from gut microbiota. As such, this tool has the potential to establish relevant metabolic interactions between diet and gut microbiota and it could potentially be the starting point for novel prediction models for diet–host–microbiota interaction.

Altogether, the novel sampling methods, the technological advances in the field of intestinal *in vivo* and *in vitro* studies, and the rapid expansion of computational-based techniques, could unlock undiscovered potentials of the small intestinal microbiota. In addition, this may lead to an expanded knowledge on diet–host–microbiota interactions, applicable for therapeutic purposes, development and screening of novel healthy food supplements or the impact of food pathogens, personalized nutrition, and the design of effective dietary strategies in cases of small intestinal dysbiosis and nutrient deficiencies.

## Concluding remarks

Over the past decade, numerous studies explored the composition and broad metabolic activities harboured within the small intestinal microbial community. For example, some food components, such as dietary fibers, are resistant to human host enzymes, hence their transformation into SCFAs, a major energy source for colonocytes, is exclusively performed by intestinal microbiota. Additionally, it emerged that distinct ecological niches exist along the small intestinal tract, shaped by the diverse physicochemical, biochemical, and physiological parameters in the duodenum, jejunum, and ileum and the nutrient availability. Despite the fact that the limited accessibility of this body site has greatly restricted for long time our knowledge on the small intestinal environment, its ecology has become a novel attractive target in food digestion and absorption research. Nevertheless, conflicting data on bacterial composition along the small intestine have limited the translation of current host–bacteria interaction knowledge into medical practice. Here, we have provided an updated overview of diverse studies aiming at characterizing the small intestinal environment and discussed how different sampling techniques, approaches, study groups, pathologies, and interindividual variabilities influences the microbial description. Additionally, as the host–microbiota interaction in the context of nutrition has become a major research target, numerous and diverse approaches have been designed for a deeper mechanistic understanding of this fine-regulated cross-talk. Here, we reviewed different *in vivo, ex vivo*, and *in vitro* approaches used to this aim, and discussed their advantages and limitations.

Along with the role of microbiota in nutrient digestion and absorption under healthy conditions, the implications for disease etiology or disease progression have also become more evident, yielding an enticing therapeutic target for nutrition-related conditions, as well. Indeed, when an extended area of the small intestine is resected, such as in SBS, or the small intestinal ecology is drastically altered, as in SIBO, the consequences for human health are severe. However, despite the numerous studies, our current knowledge on small intestinal environment and its modulation in healthy and pathological conditions, in the context of nutrition, is still limited. Novel technological advances will surely bring valuable contributions to the field. Yet, taking into consideration the strong interindividual variability, the wider translation to clinical practices needs to be carefully considered. In light of these, we believe that a personalized approach to study the dialogue between the host, microbiota, and diet in the small intestine in both healthy individuals and patients is needed to move towards novel patient-tailored therapeutic approaches with a potential improved impact on the quality of life.
